# Uptake and Population-Level Impact of Expedited Partner Therapy (EPT) on *Chlamydia trachomatis* and *Neisseria gonorrhoeae*: The Washington State Community-Level Randomized Trial of EPT

**DOI:** 10.1371/journal.pmed.1001777

**Published:** 2015-01-15

**Authors:** Matthew R. Golden, Roxanne P. Kerani, Mark Stenger, James P. Hughes, Mark Aubin, Cheryl Malinski, King K. Holmes

**Affiliations:** 1 Center for AIDS and STD,, University of Washington, Seattle, Washington, United States of America; 2 Department of Medicine, University of Washington, Seattle, Washington, United States of America; 3 Public Health-Seattle & King County, Seattle, Washington, United States of America; 4 Washington State Department of Health, Olympia, Washington, United States of America; 5 Department of Biostatistics, University of Washington, Seattle, Washington, United States of America; 6 Department of Global Health, University of Washington, Seattle, Washington, United States of America; University of Bern, SWITZERLAND

## Abstract

**Background:**

Expedited partner therapy (EPT), the practice of treating the sex partners of persons with sexually transmitted infections without their medical evaluation, increases partner treatment and decreases gonorrhea and chlamydia reinfection rates. We conducted a stepped-wedge, community-level randomized trial to determine whether a public health intervention promoting EPT could increase its use and decrease chlamydia test positivity and gonorrhea incidence in women.

**Methods and Findings:**

The trial randomly assigned local health jurisdictions (LHJs) in Washington State, US, into four study waves. Waves instituted the intervention in randomly assigned order at intervals of 6–8 mo. Of the state’s 25 LHJs, 24 were eligible and 23 participated. Heterosexual individuals with gonorrhea or chlamydial infection were eligible for the intervention. The study made free patient-delivered partner therapy (PDPT) available to clinicians, and provided public health partner services based on clinician referral. The main study outcomes were chlamydia test positivity among women ages 14–25 y in 219 sentinel clinics, and incidence of reported gonorrhea in women, both measured at the community level. Receipt of PDPT from clinicians was evaluated among randomly selected patients. 23 and 22 LHJs provided data on gonorrhea and chlamydia outcomes, respectively. The intervention increased the percentage of persons receiving PDPT from clinicians (from 18% to 34%, *p* < 0.001) and the percentage receiving partner services (from 25% to 45%, *p* < 0.001). Chlamydia test positivity and gonorrhea incidence in women decreased over the study period, from 8.2% to 6.5% and from 59.6 to 26.4 per 100,000, respectively. After adjusting for temporal trends, the intervention was associated with an approximately 10% reduction in both chlamydia positivity and gonorrhea incidence, though the confidence bounds on these outcomes both crossed one (chlamydia positivity prevalence ratio = 0.89, 95% CI 0.77–1.04, *p* = 0.15; gonorrhea incidence rate ratio = 0.91, 95% CI .71–1.16, *p* = 0.45). Study findings were potentially limited by inadequate statistical power, by the institution of some aspects of the study intervention outside of the research randomization sequence, and by the fact that LHJs did not constitute truly isolated sexual networks.

**Conclusions:**

A public health intervention promoting the use of free PDPT substantially increased its use and may have resulted in decreased chlamydial and gonococcal infections at the population level.

**Trial Registration:**

ClinicalTrials.gov NCT01665690

## Editors’ Summary

**Background:**

Every day, more than 1 million people worldwide acquire a sexually transmitted infection (STI). STIs are viral, bacterial, or parasitic infections that pass between people during sexual activity (through semen, vaginal fluids, blood, or skin-to-skin contact). Two of the most common STIs—chlamydia and gonorrhea—are caused by bacteria (*Chlamydia trachomatis* and *Neisseria gonorrhoeae*, respectively). Chlamydia rarely has any symptoms, especially in women, although some infected individuals experience pain when urinating or a discharge from the penis or vagina. The typical symptoms of gonorrhea (“the clap”) include a thick green or yellow discharge from the vagina or penis and pain when urinating, but about 10% of infected men and half of infected women have no symptoms. Both these STIs are diagnosed by testing a urine sample or a genital swab and can be treated with antibiotics. If left untreated, both infections can spread to other parts of the body and cause serious health problems including pelvic inflammatory disease (infection of the internal reproductive organs) and infertility in women.

**Why Was This Study Done?:**

Condom use during sex reduces the risk of getting or passing on chlamydia or gonorrhea, but additional approaches are needed to reduce the incidence (the number of new cases in a population in a year) of these STIs. One promising approach is expedited partner therapy (EPT)—treating the sex partners of people with a curable STI without requiring the partners to undergo a medical evaluation. EPT usually involves clinicians giving their patients antibiotics to give to their sex partners (patient-delivered partner therapy, or PDPT). Although clinical trials of EPT have shown that the approach increases partner treatment and decreases gonorrhea and chlamydia reinfection rates, EPT has not been widely adopted in the US. In this stepped-wedge, community-level randomized trial, the researchers test whether a public health program can increase the population-level use of EPT and decrease the prevalence of chlamydial infection (the proportion of a population that has chlamydia) and the incidence of gonorrhea in women. A stepped-wedge randomized trial sequentially and randomly rolls out an intervention to groups (clusters) of people and compares outcomes both between groups and within groups before and after the intervention.

**What Did the Researchers Do and Find?:**

The researchers randomly assigned local health jurisdictions in Washington State, US, to one of four study groups. The intervention, which was instituted sequentially by the groups at intervals of 6–8 months, included the provision of free PDPT packs for clinicians to hand to their patients and the provision of partner services—public health department staff offered patients free PDPT to treat up to three partners and offered to contact the partners of patients who did not want to notify their partners personally about their STI. During the 22-month trial, the intervention increased the proportion of patients receiving PDPT from clinicians from 18% to 34% and the proportion of patients receiving partner services from 25% to 45%. Chlamydia test positivity among young women attending clinics established to screen for chlamydia and gonorrhea as part of US infertility prevention efforts (sentinel clinics) decreased from 8.2% to 6.5%. The incidence of reported gonorrhea among women in the state (gonorrhea infrequently infects women in Washington State, so the researchers needed to look at a larger population than women attending sentinel clinics to get reliable figures for their analysis) decreased from 59.6 per 100,000 to 26.4 per 100,000. After adjusting for temporal trends (changes in the incidence of STIs over time unrelated to the intervention) and running appropriate statistical models, the researchers estimated that the intervention was associated with an approximately 10% reduction in chlamydia positivity and gonorrhea incidence, although these results were statistically insignificant and may therefore be chance findings.

**What Do These Findings Mean?:**

Some aspects of this trial may limit the accuracy and robustness of its findings. For example, during the study period, there was an increase in partner services outside the trial that might have contributed to the observed decreases in STIs and decreased the study’s ability to find an effect associated with the study intervention. Nevertheless, these findings show that the implementation of a public health program promoting the provision of free PDPT substantially increased PDPT use by clinicians in Washington State. Moreover, although the trial did not definitively show that EPT decreased chlamydia test positivity or the incidence of gonorrhea, substantial population-level declines in both STIs occurred concurrently with the introduction of the intervention. The researchers conclude that the continued use and expansion of EPT as a way to reduce the incidence of chlamydia and gonorrhea is warranted, and suggest that the public health intervention used in this trial provides a model for health departments seeking to increase EPT use.

**Additional Information.:**

Please access these websites via the online version of this summary at http://dx.doi.org/10.1371/journal.pmed.1001777.

- The World Health Organization provides a fact sheet about sexually transmitted infections (in several languages)
- The US Centers for Disease Control and Prevention provides detailed information about sexually transmitted diseases, chlamydia, gonorrhea, and expedited partner therapy (some information available in several languages)
- The UK National Health Service Choices website provides information about chlamydia (including some personal stories) and gonorrhea
- Healthtalkonline (a not-for-profit organization) provides personal stories about sexually transmitted infections
- MedlinePlus provides links to further resources about sexually transmitted infections (in English and Spanish)
- More information about this trial is available
- The Washington State Department of Health website includes information about Washington State’s EPT program, including EPT guidelines and information related to the legal process for instituting EPT in the state

## Abstract 3

Matthew Golden and colleagues report on the effects of promoting treatment without testing for sexual partners of patients with chlamydia or gonorrhea.

## Introduction

*Chlamydia trachomatis* and *Neisseria gonorrhoeae* cause the two most commonly reported infections in the United States [1], and new approaches to preventing these sexually transmitted infections (STIs) are needed.

Increasing the number of exposed sex partners of persons with gonorrhea or chlamydial infection who receive treatment could decrease the incidence and prevalence of infection. However, few US health departments currently devote substantial resources to partner services for bacterial STIs other than syphilis, and over 80% of patients with gonorrhea or chlamydial infection are left to notify their partners without assistance [2]. Randomized controlled trials conducted in the late 1990s and early 2000s found that expedited partner therapy (EPT)—the practice of treating the sex partners of persons with curable STIs without requiring the partners to first undergo a medical evaluation—increases partner treatment and decreases rates of reinfection with gonorrhea or chlamydia [3–6]. In the wake of these trials, many US states made EPT legal, and the practice is now lawful in most of the country [7]. In most instances, EPT involves medical practitioners giving patients medication to give to their sex partners, a practice called patient-delivered partner therapy (PDPT).

Many physicians at least sometimes give patients medication to give to a sex partner [8–10], but relatively few persons with gonorrhea in the US currently receive PDPT [11], and the proportion of patients with chlamydial infection who receive PDPT is not well defined. Working in King County, Washington (WA), we previously reported that a population-based program could significantly increase medical providers’ use of PDPT [12]. However, the scale of PDPT promotion and evaluation in King County has been an exception, and most efforts to institute and assess PDPT programs have focused on individual clinics or small groups of clinics [13–16]. The feasibility of promoting EPT more widely and the intervention’s population-level effect are unknown.

We conducted a stepped-wedge, community-level randomized trial to test the hypothesis that a public health program could increase the population-level use of EPT, and through this effort decrease the prevalence of chlamydial infection and incidence of gonorrhea in women. Stepped-wedge trials are a type of cluster randomized trial in which clusters of clinics or communities receive an intervention in a randomly assigned order; outcome analyses then compare variance both between communities and within a community before and after the intervention. We have previously described this study design and its use in the context of this community-level trial [17]. The study employed cluster randomization in order to measure the population-level impact of our intervention, and used a stepped-wedge design because it allowed all areas of the state to eventually adopt the intervention, which promotes the use of EPT, a clinically beneficial intervention.

## Methods

### Ethics Statement

The University of Washington Institution Review Board approved study procedures. Local health officers in WA State agreed to their jurisdictions’ participation in the trial. Because the trial did not enroll individuals as part of a study, persons receiving interventions through the study did not undergo a consent process.

### Study Design and Participating Communities

Local health jurisdictions (LHJs)—administrative units that usually correspond to a single county—were the study’s unit of randomization and analysis. All WA State LHJs except King County were eligible to participate in the trial. We excluded King County because the study intervention was developed there and could not practically be discontinued and then restarted [18]. The trial initiated the study intervention in groups of LHJs (i.e., waves) at four time points (i.e., steps) separated by intervals of 6–8 mo between October 2007 and August 2009. A computer randomly assigned LHJs into the four waves, with randomization stratified by region and LHJ size. The allocation of LHJs into waves was concealed from state and local public health department staff and from all investigators except the study biostatistician until 1 mo before initiation of the intervention in each wave. No blinding was done after the study intervention was initiated.

### Intervention

The intervention was designed to increase the treatment of sex partners of individual heterosexual patients and included two components: (1) promotion of PDPT use and (2) targeted provision of public health partner services. In each LHJ, the study sought to increase PDPT use by supplying free PDPT packs to clinicians and by making free packs available through commercial pharmacies for clinicians to prescribe for their patients’ partners. When the intervention was initiated in each LHJ, the research team and local health department staff sent a letter to every clinician who had reported one or more cases of bacterial STI in the prior year. (Clinicians treating STIs in WA State include a diverse array of physicians [family medicine practitioners, internal medicine practitioners, obstetricians and gynecologists] and mid-level practitioners.) This letter informed clinicians that state guidelines recommend that medical providers offer PDPT to all heterosexual individuals with gonorrhea or chlamydial infection when the clinician cannot “otherwise assure” that all of a patient’s potentially exposed partners will be treated [19]. The letter also included information about how to obtain free PDPT to stock in their office or clinic, and how to prescribe free PDPT through local pharmacies.

In each LHJ, research team and public health staff members visited clinics and offices that had reported large numbers of cases of gonorrhea or chlamydial infection in the year prior to program initiation; these clinics were chosen in collaboration with local staff and were not defined using fixed criteria. During these visits, public health staff supplied clinics with PDPT packets and educated clinic staff and medical providers about the program.

Although all clinicians were eligible to receive free PDPT packs to stock in their offices or clinics, the study also developed a mechanism for medical providers to prescribe free PDPT packs through local commercial pharmacies. Only selected pharmacies stocked PDPT packs for use as part of the study. These pharmacies typically belonged to one of several large commercial chains. The study chose participating pharmacies in collaboration with local public health staff in each LHJ with the goal of assuring widespread access to PDPT packs. A total of 157 pharmacies participated in the study.

Additional efforts to promote PDPT use in intervention communities included annual letters to medical providers reporting at least one case of gonorrhea or chlamydial infection, letters to medical providers whose patients received partner services but not PDPT informing them about the program, information on STD case report forms outlining WA State’s PDPT guidelines and how to obtain free PDPT, discussions with clinic or office staff during telephone calls to complete incomplete case reports, and inclusion of information about PDPT in continuing medical education trainings. The study also worked with the largest health maintenance organization in the state and with large family planning organizations to promote PDPT use. All medications for PDPT were distributed in premade packs that met state legal requirements and included condoms, information about STIs, and a medication allergy warning in English and Spanish that asked recipients to call the study coordinator if they experienced an adverse drug reaction. All packs included 1 g of azithromycin; packs for gonorrhea also included a 400-mg dose of cefixime. Both medications were to be taken as a single dose.

The study sought to target the provision of partner services to persons at high risk for failing to ensure a partner’s treatment. Diagnosing medical providers and laboratories in WA State are required to report gonorrhea or chlamydial infection cases to the local and state public health department. Medical providers do this using a case report form. As part of the trial, we modified that form to ask providers to indicate whether they wanted the public health department to provide their patient with partner services. The form recommended that providers refer patients with any of the following risks for partner services: (1) >1 sex partner in the 60 d prior to diagnosis, (2) ≥1 sex partner in the prior 60 d whom the patient did not anticipate having sex with again, (3) the patient indicated he/she could not or would not notify a partner, or (4) the patient was a man who has sex with men. Prior studies have associated the first two of these factors with the failure of partners to receive treatment [12,18].

Partner services consisted of a structured interview that guided public health staff (usually disease intervention specialists) to define a partner treatment plan for a patient’s most recent partner and all partners from the prior 60 d. Staff providing partner services offered patients free PDPT to treat up to three partners, and offered to directly contact partners whom patients did not want to notify themselves. When contacting partners, staff advised them to seek medical evaluation, but also offered them treatment without such an evaluation. Both patients and partners could obtain free PDPT through commercial pharmacies or via the mail. The study intervention did not include efforts to find index cases or partners through field investigations (e.g., going to persons’ homes or places of work).

Although the study sought to phase in partner services according to a prespecified design, in 2007 the WA State legislature allocated new funds for communicable disease that some LHJs used to increase partner services in 2008 and 2009, a change beyond the control of the study. The study did not measure which LHJs increased the provision of partner services using new state funding. No public health department funds were used to purchase or promote the use of PDPT outside of the study’s randomization sequence.

### Study Outcomes and Statistical Methods

The study’s two primary, prespecified outcomes measured for each LHJ were (1) chlamydia test positivity among women ages 14–25 y tested through Infertility Prevention Project (IPP) or Planned Parenthood of Western Washington clinics and (2) the incidence of reported gonorrhea in women. Prespecified secondary outcomes included use of PDPT by medical providers, adverse reactions, and recurrent gonorrhea and recurrent chlamydial infection; these last two outcomes will be presented in a separate report.

The study’s chlamydia positivity endpoint used results for all women ages 14–25 y tested for *C. trachomatis* in IPP clinics, including tests performed in the Planned Parenthood of Western Washington laboratory, during five prespecified time periods (analysis intervals; 7/1/07–9/30/07, 3/1/08–5/31/08, 10/15/08–1/14/09, 5/1/09–7/31/09, 6/1/10–8/31/10). These intervals correspond to the last 3 mo of data in each intervention period (when the intervention should have reached maximal effect). The IPP was a national chlamydia testing program funded by the Centers for Disease Control and Prevention (CDC), and data from the IPP have previously been used to monitor chlamydial prevalence in young women [20–22]. During the study period, IPP testing occurred in 219 clinics in WA State, including 98 family planning, women’s health, or reproductive health clinics; 36 community clinics; 29 STD or HIV testing clinics; 17 adolescent or school-based clinics; and 39 clinics of other types. In 2008, Planned Parenthood of Western Washington began testing women with health insurance at a separate laboratory, while continuing to send specimens from uninsured women to the IPP laboratory. Because this change could have altered the IPP population, the study included tests performed in both the Planned Parenthood of Western Washington and the IPP laboratories in the outcome analysis. (Throughout the paper we refer to the clinics providing chlamydia outcome data as IPP clinics, which includes the Planned Parenthood of Western Washington laboratory.) All chlamydial testing was done using nucleic acid amplification tests.

The outcome analyses focused on women because men are not typically screened for STIs—meaning that no sentinel population of tested men existed in the state—and because reported STI incidence in men includes outcomes in both heterosexual men and men who have sex with men (MSM); CDC and WA State guidelines recommend against the use of EPT in MSM [23], and our study did not seek to increase EPT use in MSM. We elected to use chlamydia positivity in sentinel populations of women as our outcome because we did not believe that testing a random sample of the entire state population of young women would be feasible, and believed that positivity in a describable population would be a better outcome than incidence of reported infections, which reflects both testing patterns and true incidence. Because gonorrhea has become an infrequent infection in women in WA State, it was not possible to power the study to use gonorrhea positivity within IPP clinics as an outcome. As a result, we used the incidence of reported gonorrhea in women as our gonorrhea outcome.

The primary analysis of the study’s chlamydia outcome used a mixed effects generalized linear model with a log link and included covariates for intervention status andtime period; random effects for LHJ and clinic within LHJ were used to adjust for the fact that not all clinics contributed to the IPP database during all time periods. The study design assumed 162 women testing in each of 24 LHJs in each analysis interval, 5% chlamydia positivity, a coefficient of variation between LHJs of 0.33 (based on preliminary data), and α = 0.05 (two-tailed). We anticipated 80% power to detect a prevalence ratio of 0.70 [17]. The effect size anticipated was consistent with preliminary mathematical modeling work undertaken in preparing the study grant application. The study gonorrhea outcome was the number of cases of gonorrhea reported among women in each participating LHJ in each prespecified time period. This analysis used a marginal Poisson regression model with covariates for intervention status and time period, an offset for female population size within the LHJ, clustering by LHJ, and an exchangeable working correlation structure. Robust variances are reported. These analysis models were prespecified. The study results also include a single post hoc analysis assessing the intervention’s impact on a combined gonorrhea and chlamydia endpoint. This analysis was similar to the analysis of gonorrhea but included two outcomes for each LHJ for each time period: (1) gonorrhea cases, as described above, and (2) chlamydia cases from the IPP clinics with offset equal to the number of women tested in the LHJ during the analysis interval. The model included separate time trends for each disease but a common intervention effect. All analyses were carried out using Stata version 12.1 (StataCorp, College Station, Texas).

In order to assess the comparability of the population of women in which we evaluated our *C. trachomatis* outcome to all sexually active women in the communities participating in the trial, we compared the age and race/ethnicity distributions of women tested in IPP clinics to US census data for women in the WA State LHJs included in the study. Because many younger women are not sexually active, and consequently would not be at risk for STIs or tested for *C. trachomatis*, we used US national data from the National Survey of Family Growth 2006–2010 [24] to define the proportion of women at each age who have ever had sex, and multiplied the number of women in each age group by that proportion to define the number of sexually active women in each age group in the state. The proportion of black versus white young women who were sexually active in the National Survey of Family Growth varied by only 3%, and therefore we did not adjust our statewide population estimate for race/ethnicity.

The study was also designed to assess the scalability of a public health EPT intervention. This effort initially included measurement of four outcomes: (1) the proportion of persons receiving PDPT from their medical provider, (2) the proportion of persons receiving public health partner services, (3) the proportion of persons receiving either PDPT from their medical provider or partner services from public health staff, and (4) the proportion of persons receiving PDPT from either their medical provider or from public health partner services staff. In September 2009, we added a question to routine partner services interviews to measure the proportion of persons who were offered PDPT by their medical provider, a fifth outcome related to the intervention’s uptake; data on this final outcome were not available for the pre-intervention periods in study waves 1–3, but were available for at least part of the intervention period for all four study waves. As a result, we report this outcome only for the intervention periods. To measure these outcomes, the study attempted to interview random samples of patients comprising 20% of reported cases of gonorrhea and 20% of reported cases of chlamydial infection; random samples were automatically drawn by the public health department computer case registry as cases were entered into that system. Staff interviewed patients starting 4–6 mo prior to each wave’s initiation of the intervention and throughout the intervention period; interviewers made a minimum of three attempts to contact each selected interviewee by telephone. Staff did not attempt field visits to interview patients. The analysis of PDPT use excluded MSM, who were identified using information collected on the case report and during partner services interviews. Of 9,690 randomly selected women and men not identified as MSM, 5,741 (59%) had complete interview data. The median time from patient treatment to interview was 11 d (interquartile range 5–22 d), and 92% of interviews were conducted by telephone. To adjust for nonresponse, estimates of the proportion of persons offered and receiving PDPT from their medical provider used post-stratification weights based on gender, race/ethnicity, STI diagnosis, and, during the intervention period, whether providers referred patients for partner services. Confidence intervals for proportions were computed on the logit scale and backtransformed using standard methods for random samples, adjusting for the weighting. To estimate the proportion of persons who received PDPT from either their provider or from public health staff, we summed the estimated proportion receiving PDPT from medical providers with the directly measured proportion of all cases in patients who were not MSM who received PDPT from public health staff and who did not also receive PDPT from their medical provider.

Adverse drug reactions were ascertained based on calls to the study coordinator; information sheets included in each partner pack asked recipients to call the coordinator if they experienced a drug reaction. The study did not conduct follow-up interviews with patients to ascertain the effect of the intervention on partnership dissolution or intimate partner violence.

The trial’s sponsor did not require it to be registered in ClinicalTrials.gov, and the registration website was not designed to accommodate information on community-level trials that do not enroll individuals. Consequently, we did not register the trial prior to the study’s initiation. We did register the study in ClinicalTrials.gov after study initiation.

We are not aware of other ongoing community-level trials related to EPT, though several individual-level trials have evaluated the effect of EPT (ClinicalTrials.gov NCT01720654; [3–5,25,26]).

## Results

### Populations Participating in the Trial

**Figure 1:**
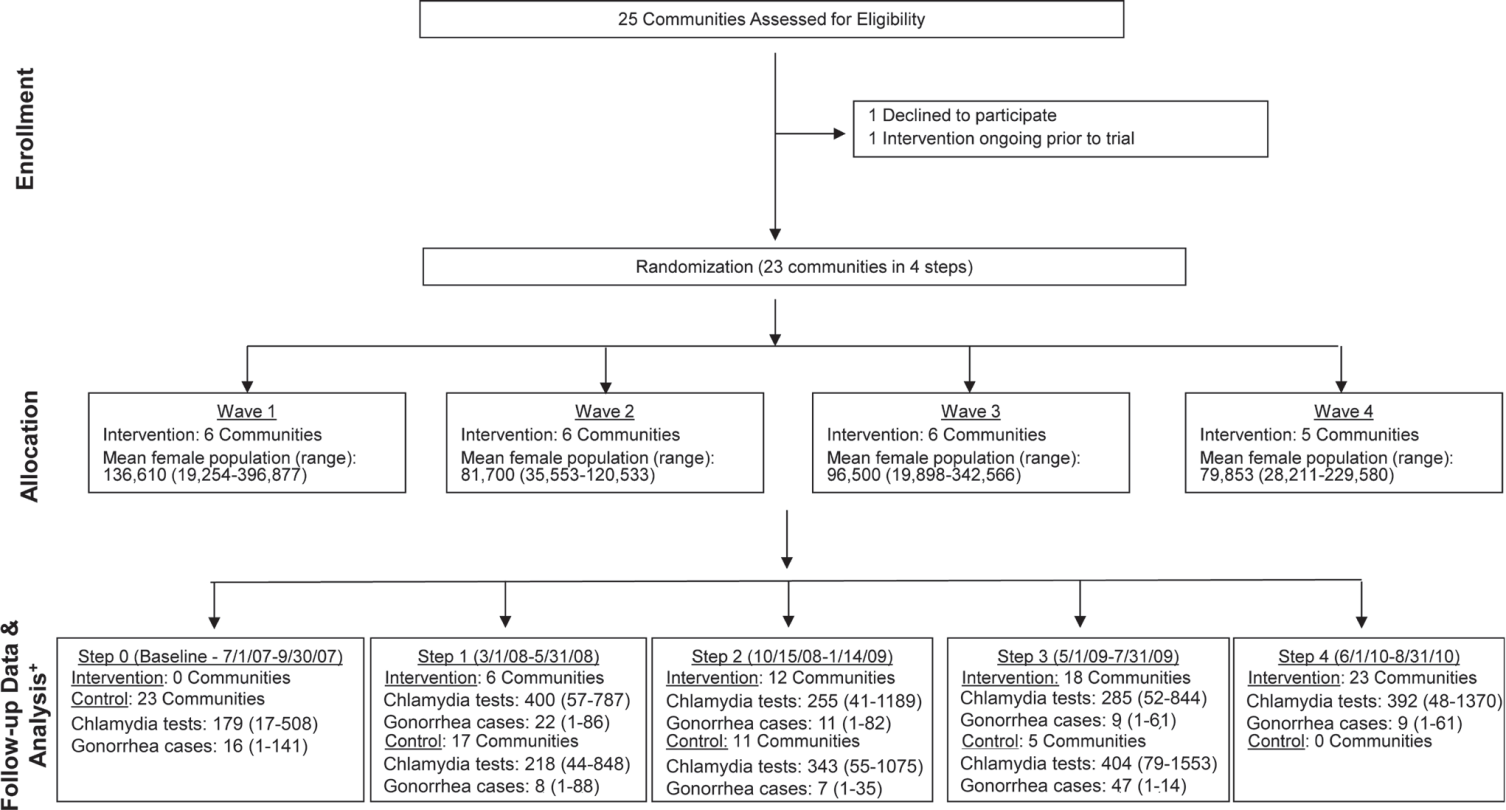
Study flow diagram. Modified for stepped-wedge design from suggested CONSORT criteria format for cluster randomized trials [47]. ^+^Numbers of tests and cases presented as means with ranges.

**Figure 2:**
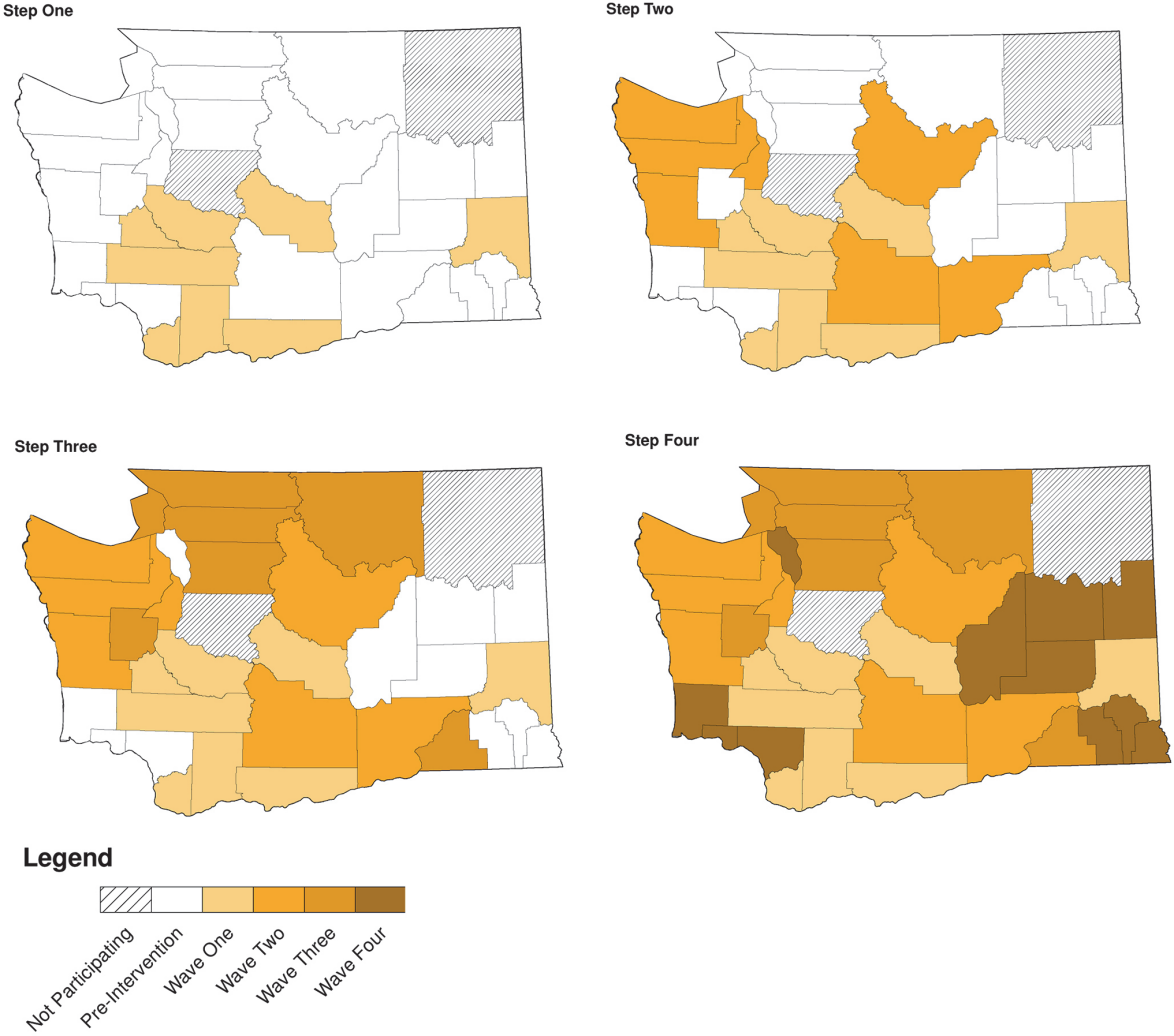
Washington State local health jurisdictions instituting an expedited partner therapy intervention in four temporally defined waves.

Of WA State’s 25 LHJs, 23 participated in the study (Figs. 1 and 2). Table 1 presents data from clinics participating in the WA State IPP program in the year prior to the start of the study; these clinics include all clinics in which we measured chlamydia positivity, one of our main study outcomes, and is stratified by study wave. Chlamydia outcome data were available at baseline and for each of the four steps for 22 of the 23 participating LHJs (one LHJ had no clinical site providing data). Table 2 compares the age, race, and ethnicity of women tested for chlamydial infection in clinics participating in the IPP program with those of women ages 14–25 y in the areas of WA State participating in the study. Women tested at sentinel clinical sites were younger than the overall population of sexually active women in the areas served by the clinics. The study had data on the incidence of newly diagnosed and reported gonorrhea for all LHJs at each step.

**Table 1:** Study population size and characteristics in local health jurisdictions in each study wave during the year prior to the intervention (October 2006–September 2007).

**Population Characteristic**	**Wave 1 Counties**	**Wave 2 Counties**	**Wave 3 Counties**	**Wave 4 Counties**	**Total**
**Female population size**	820,033	495,020	583,866	401,427	2,300,346
**Number women ages 14–25 y**	143,091	80,776	97,661	69,147	390,675
**Gonorrhea cases/rate per 100,000**	620/76	197/40	240/41	236/58	1,293/56
**Number of chlamydia tests performed in women ages 14–25 y through IPP**	6,563	5,020	3,398	2,928	17,909
**Chlamydia positivity (percent)**	535 (8.2%)	402 (8.1%)	239 (7.1%)	209 (7.2%)	1,385 (7.8%)
**Number of IPP clinics**	26	29	25	17	97
**Mean (SD) age, years**	20.4 (2.6)	20.0 (2.8)	19.4 (2.8)	19.7 (2.7)	20.0 (2.7)
**Race, number (percent)^a^**					
White	3,653 (83.4%)	2,395 (68.3%)	1,819 (86.2%)	1,846 (91.2%)	9,713 (80.8%)
Black	335 (7.6%)	84 (2.4%)	68 (3.2%)	70 (3.5%)	557 (4.6%)
Native American	91 (2.1%)	105 (3.0%)	96 (4.6%)	50 (2.5%)	342 (2.8%)
Asian	192 (4.4%)	37 (1.0%)	59 (2.8%)	33 (1.6%)	321 (2.7%)
Other	230 (5.2%)	915 (26.1%)	107 ((5.1%)	59 (2.9%)	1,311 (10.9%)
**Hispanic ethnicity, number (percent)**	764 (12.7	1,638 (33.5%)	400 (12.1%)	244 (8.4%)	3,046 (17.8%)
**Reason for visit, number (percent)^b^**					
Routine	2,790 (43.7%)	2,616 (52.9%)	1,452 (43.5%)	1,088 (37.8%)	7,946 (45.3%)
Symptoms	1,206 (18.5%)	555 (11.1%)	502 (14.8%)	636 (21.8%)	2,899 (16.3%)
STD screening	3,120 (48.9%)	2,493 (50.4%)	2,110 (63.2%)	1,422 (49.4%)	9,145 (52.1%)
Exposed to *C. trachomatis*	142 (2.2%)	74 (1.5%)	100 (3.0%)	88 (3.1%)	404 (2.3%)
Exposed to other STI	55 (0.9%)	42 (0.8%)	52 (1.6%)	30 (1.0%)	179 (1.0%)
Pregnancy-related	800 (12.5%)	909 (18.4%)	365 (10.9%)	237 (8.2%)	2,311 (13.27%)
Rescreen	174 (2.7%)	117 (2.4%)	98 (2.9%)	82 (2.8%)	471 (2.7%)
**Risk, number (percent)^c^**					
History of chlamydia	456 (7.2%)	276 (5.6%)	225 (6.8%)	179 (6.6%)	1,136 (6.6%)
≥2 partner	626 (10.1%)	405 (8.2%)	374 (11.4%)	341 (12.5%)	1,746 (10.2%)
New sex partner	1,442 (23.1%)	949 (19.2%)	816 (24.6%)	736 (27.1%)	3,943 (22.9%)
Symptomatic partner	287 (5.4%)	137 (3.0%)	122 (3.9%)	108 (4.2%)	654 (4.2%)
**Condoms used during last sexual activity, number (percent)**	1,359 (23.6%)	982 (20.0%)	712 (21.6%)	613 (23.6%)	3,666 (22.1%)

The population of each wave includes persons residing in the LHJs that initiated the study intervention as part of that wave.

^a^ *n* = 4,379, 3,508, 2,111, and 2,024 non-missing responses for race for waves 1–4, respectively. Race and ethnicity defined based on medical provider report using a standardized reporting record.

^b^Patients could report more than one reason for visit.

^c^Chlamydia diagnosis in prior year, and ≥2 sex partners, a new sex partner, or a symptomatic partner in the prior 60 d.

SD, standard deviation.

**Table 2:** Characteristics of women tested for *C. trachomatis* in clinics providing outcome data for the trial compared to all women in areas of WA State participating in the trial.

**Characteristic**	**Estimated Population of Women Who Had Ever Had Sex with a Man in WA State LHJs Participating in the Trial, 2008^a^**	**Women Tested for Chlamydial Infection in Sentinel Clinical Sites, 2007–2010**
**Number of women ages 14–25 y**	243,666	91,971^b^
**Age group**		
14–17 y	12.4%	17,359 (18.9%)
18–20 y	28.4%	40,159 (43.7%)
21–23 y	35.4%	28,522 (31.0%)
24–25 y	23.7%	5,931 (6.5%)
**Race**		
White	87.3%	65,516 (71.2%)
Black	4.0%	3,214 (3.5%)
Native American	2.7%	1,820 (2.0%)
Asian	6.0%	3,108 (3.4%)
Other	Not measured	9,337 (10.2%)
**Hispanic ethnicity**	15.1%	13,375 (14.7%)

Data are percent or number (percent).

^a^Estimates of sexually active women were calculated by defining the proportion of women at each age who have ever had sex in the US national data from the National Survey of Family Growth 2006–2010, and multiplying the number of women in each age group by that proportion.

^b^De-duplicated; each woman contributes only one record. Data on race were missing for 9.7% of women tested in sentinel clinical sites.

### Intervention Uptake

**Figure 3:**
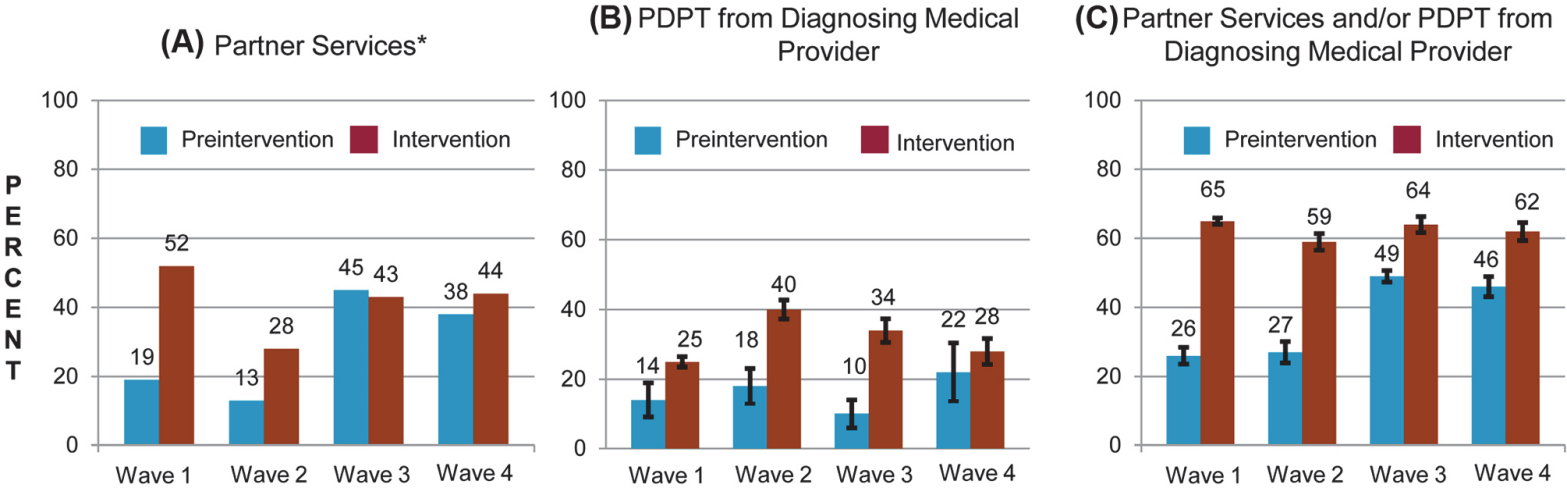
Percentage of persons with gonorrhea or chlamydial infection who received components of the study intervention in periods before and during the study intervention, by study wave. Percentage of persons receiving (A) PDPT from their diagnosing clinician, (B) public health partner services, or (C) either PDPT or public health partner services. *The percentage of persons receiving partner services was directly measured and is not an estimate. Consequently, there are no confidence intervals on data for this outcome.

Between May 2007 and December 2010, participating LHJs reported 5,164 and 52,945 cases of gonorrhea and chlamydial infection, respectively, excluding cases in MSM. The percentage of cases of gonorrhea or chlamydial infection in patients who were not MSM across all study waves who received partner services increased from 25.2% in the pre-intervention periods to 44.9% during the intervention periods (*p* < 0.001), and this increase was evident in diverse groups defined by demographic factors and sources of care (Fig. 3; Table 3). However, this increase was apparent only in the first two study waves that instituted the intervention, before the impact of new, unanticipated state funding for partner services was fully realized.

**Table 3:** Receipt of public health partner services within subgroups of patients during baseline and intervention periods.

**Population Characteristic**	**Baseline**			**Intervention**		
	**Total**	***N***	**Percent**	**Total**	***N***	**Percent**
**Total**	5,974	1,506	25.2%	39,234	17,614	44.9%
**Age**						
14–19 y	1,868	497	26.6%	12,281	5,723	46.6%
20–24 y	2,255	543	24.1%	15,335	6,938	45.2%
25–29 y	1,014	255	25.2%	6,526	2,816	43.2%
30–34 y	410	122	29.8%	2,580	1,079	41.8%
35–44 y	285	70	24.6%	1,801	751	41.7%
>45 y	85	15	17.7%	543	252	46.4%
**Race**						
White	2,951	817	27.7%	19,513	8,812	45.2%
Black	532	163	30.6%	3,708	1,940	52.3%
Native American	193	44	22.8%	1,045	355	34.0%
Asian	124	43	34.7%	1,140	537	47.1%
Other	260	42	16.2%	2,148	897	41.8%
Multiple	108	34	31.5%	680	250	51.5%
Unknown	1,806	363	20.1%	11,000	4,723	42.9%
**Ethnicity**						
Hispanic	910	179	19.7%	5,626	2,033	36.1%
Non-Hispanic	2,927	855	29.2%	19,924	9,270	46.5%
Unknown	2,137	179	22.1%	13,684	6,311	46.1%
**Gender**						
Female	4,457	1,139	25.6%	29,885	13,645	45.7%
Male	1,470	364	24.8%	9,310	3,959	42.5%
**Diagnosis**						
Chlamydia only	5,248	1,227	23.4%	36,054	15,907	44.1%
Gonorrhea only	462	172	37.2%	2,122	1,131	53.3%
Both	264	107	40.5%	1,058	576	54.4%
**Provider type**						
Family planning	1,441	385	26.7%	8,678	3,513	40.5%
Women’s health	487	141	29.0%	3,626	1,691	46.6%
Private	1,319	358	27.1%	9,923	4,814	48.5%
ER/urgent care	531	149	28.1%	3,978	2,138	53.8%
Military	385	26	6.8%	3,730	1,703	45.7%
Other	1,211	310	25.6%	7,709	3,157	41.0%
Unknown	600	137	22.8%	1,590	598	37.6%
**IPP clinics^a^**						
IPP clinic	1,747	517	29.6%	10,817	4,435	41.0%
Not IPP clinic	4,226	989	23.4%	28,409	13,177	46.4%

^a^Clinics that provided *C. trachomatis* outcome data.

ER, emergency room.

The study intervention significantly increased the use of PDPT by diagnosing medical providers. As was the case for the increase in partner services, this increase occurred across subgroups defined by demographic factors, STI diagnosis, and type of medical provider (Table 4). Over the study period, public health and research staff distributed 19,299 PDPT packs to 472 different medical clinics, offices, and pharmacies. Pharmacies dispensed 7,110 PDPT packs. (The study measured medication dispensing only through pharmacies; the number of packs dispensed by medical provider offices was not measured.) Based on interviews with randomly selected patients, the percentage of patients receiving PDPT from their diagnosing provider increased from 18.3% in the pre-intervention periods to 34.0% during the intervention periods (*p* < 0.001). Including PDPT prescribed by either medical providers or by public health staff providing partner services, the estimated proportion of patients who received PDPT increased from 18.3% in the pre-intervention periods to 43.9% in the intervention periods. In 2010, the final year of the study, providers offered PDPT to an estimated 38.1% and 51.9% of all heterosexual individuals with gonorrhea and chlamydial infection, respectively, and 31.8% and 44.7% of patients received PDPT from either their medical provider or from public health staff providing partner services. Among 1,670 persons randomly selected for study interviews who reported receiving PDPT from a medical provider during the intervention period, 1,550 (92.6%) stated that they gave a PDPT pack to at least one sex partner. Providers offered PDPT more frequently to patients who they did not refer for partner services than to those they did refer for such services (80.9% versus 29.3% *p* < 0.001), and patients who did not receive partner services were much more likely to receive PDPT from medical providers than those who received partner services (62.5% versus 12.0%, *p* < 0.001). Persons referred for partner services were more likely to have >1 sex partner or a partner they did not anticipate having sex with again in the future, our criteria for referral for public health partner services (32.4% versus 20.1%, *p* < 0.001).

**Table 4:** Medical providers’ use of patient delivered partner therapy within subgroups of patients during baseline and intervention periods.

**Population Characteristic**	**Baseline**				**Intervention**			
	**Total**	***N***	**Weighted Percent^a^**	**95% CI^b^**	**Total**	***N***	**Weighted Percent^a^**	**95% CI^b^**
**Total^c^**	580	110	18.3%	15.3–21.7	4,701	1,598	34.0%	32.6–35.4
**Age**								
14–19 y	199	41	20.5%	15.5–26.7	1,509	510	33.9%	31.5–36.3
20–24 y	205	41	19.1%	14.2–25.2	1,851	652	35.2%	33.0–37.4
25–29 y	95	13	13.0%	7.5–21.6	775	265	34.2%	31.0–37.6
30–34 y	50	10	19.2%	10.3–33.0	299	104	34.8%	32.2–37.5
35–44 y	22	3	12.9%	3.7–36.4	185	55	29.5%	23.2–36.6
>45 y	6	0	0%	—	67	9	13.3%	6.9–24.0
**Race**								
White	331	74	22.2%	18.0–27.0	2,465	913	37.3%	35.4–39.2
Black	55	6	10.0%	4.3–21.5	446	101	22.7%	19.0–26.8
Native American	14	4	29.0%	9.8–60.5	96	36	37.4%	28.2–47.6
Asian	18	2	10.8%	2.2–38.9	143	65	45.5%	37.4–53.8
Other	22	2	8.7%	1.9–32.3	255	120	47.0%	41.0–53.1
Multiple	16	3	17.7%	4.8–47.8	102	40	39.5%	30.4–49.4
Unknown	124	19	15.1%	9.7–22.7	1,194	323	27.0%	24.6–29.6
**Ethnicity**								
Hispanic	140	31	21.5%	15.4–29.2	958	418	43.9%	40.7–47.1
Non-Hispanic	366	70	18.3%	14.7–22.5	3,343	1,074	32.0%	30.4–33.6
Unknown	74	9	12.4%	6.5–22.3	400	106	26.7%	22.6–31.2
**Gender**								
Female	456	104	22.6%	19.0–26.6	3,704	1,437	39.2%	37.6–40.8
Male	124	6	4.7%	2.1–10.3	997	161	16.6%	14.4–19.0
**Diagnosis**								
Chlamydia only	515	106	19.8%	16.6–23.4	4,333	1,523	35.2%	33.8–36.6
Gonorrhea only	55	2	3.6%	0.7–16.2	253	56	22.1%	18.4–26.4
Both	21	2	8.8%	1.8–33.3	115	19	16.0%	10.4–23.9
**Provider type**								
Family planning	170	49	28.7%	22.3–36.1	1,198	640	53.5%	50.7–56.3
Women’s health	51	12	23.9%	13.9–37.9	454	212	47.4%	42.8–52.0
Private	113	16	13.7%	8.5–21.4	1,216	381	31.3%	28.8–34.0
ER/urgent care	49	3	5.4%	1.6–16.9	507	54	10.6%	8.2–13.6
Military	16	2	10.7%	2.0–41.8	329	47	14.3%	10.9–18.5
Other	120	16	12.2%	7.3–19.7	836	208	24.9%	22.0–28.0
Unknown	61	12	19.3%	11.1–31.5	161	56	35.3%	28.2–43.1
**IPP clinics^d^**								
IPP clinic	214	59	26.9%	21.4–33.2	1,457	728	49.9%	47.4–52.5
Not IPP clinic	366	51	13.4%	10.3–17.2	3,244	870	26.8%	25.3–28.4

^a^Estimated based on interviews with randomly sampled, interviewed patients.

^b^Confidence interval accounts for weighting; computed on logit scale and backtransformed.

^c^The magnitude of the intervention effect did not vary significantly for any of the subgroups defined in the table.

^d^Clinics that provided *C. trachomatis* outcome data.

ER, emergency room.

### Effect of the Intervention on Chlamydia Positivity and Reported Gonorrhea Incidence in Women, and Adverse Effects

**Figure 4:**
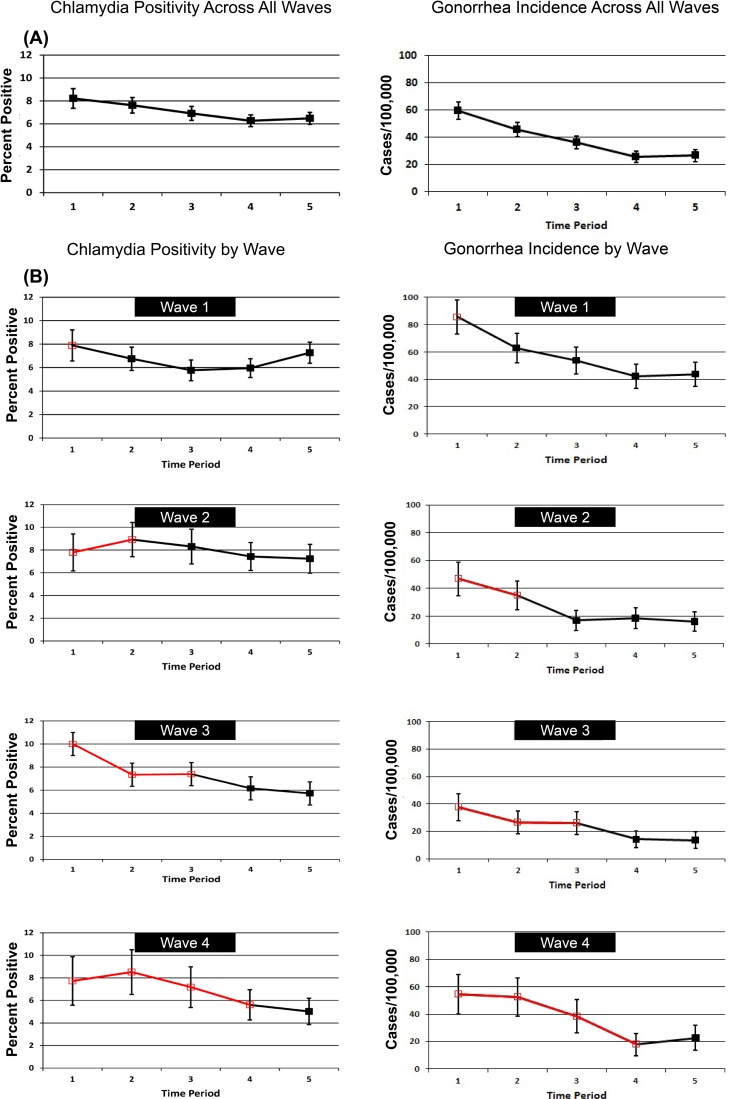
Trends in chlamydia test positivity and gonorrhea incidence for 2007–2010 among women in 23 local health jurisdictions in Washington State. Chlamydia test positivity and gonorrhea incidence (A) across all waves and (B) by wave. Open symbols and red lines indicate measurement and time before the institution of the study intervention, and solid symbols and black lines represent intervention time periods. Time periods are 3-mo analysis periods occurring prior to initiation of the intervention in each wave.

Fig. 4 shows patterns of chlamydia positivity and gonorrhea incidence overall (Fig. 4A) and in each wave of LHJs receiving the intervention (Fig. 4B), with red lines and open symbols representing pre-intervention time periods, and black lines and solid symbols representing intervention time periods. Chlamydia positivity among women ages 14–25 y tested in IPP clinics in LHJs participating in the trial decreased over the study period from 8.2% to 6.5% (*p* < 0.001), while the annualized incidence of gonorrhea in women declined dramatically from 59.6 to 26.4 per 100,000 (*p* < 0.001). After adjusting for temporal trends, the intervention was associated with an approximately 10% reduction in both chlamydia positivity and gonorrhea incidence, though the confidence bounds on these outcomes both included one (Table 5). A post hoc analysis that combined the study’s two primary outcomes resulted in narrower confidence bounds, suggesting that the intervention was associated with at least a small population-level effect. The estimated coefficient of variation between LHJs for chlamydia positivity was 0.24 and for gonorrhea incidence was 0.40, close to the estimate used for the study’s initial power calculations (0.33).

**Table 5:** Association of the study intervention with chlamydia test positivity and reported gonorrhea incidence in women.

**Study Outcome**	**Prevalence/Rate Ratio (95% CI)**	***p*-Value**
Chlamydia positivity in women ages 14–25 y	0.89 (0.77–1.04)	0.15
Reported gonorrhea incidence in women	0.91 (0.71–1.16)	0.45
Combined chlamydia positivity and gonorrhea incidence	0.90 (0.80–1.01)	0.06

No episodes of anaphylaxis or other major adverse drug reactions were reported to the study coordinator during the trial. The study did not record the number of instances of nausea or vomiting associated with study medications.

## Discussion

We found that a public health intervention could significantly increase both medical providers’ use of PDPT and the targeted provision of partner services in a state with a population over 6.5 million. Chlamydial test positivity among women evaluated in sentinel clinics and the incidence of gonorrhea among women significantly declined during the study period, and although the confidence bounds for our primary study outcomes included one, the relative risk and rate ratios associated with the study intervention were consistent with the intervention resulting in an approximately 10% reduction in our population-level measures of gonorrhea and chlamydial infection in women.

We believe that the best interpretation of our findings is that our intervention likely resulted in a modest reduction in STI morbidity. Our study was powered to identify a 30% reduction in chlamydia positivity and gonorrhea incidence, effect sizes that substantially exceeded the 10% reduction observed. In a post hoc analysis that combined gonorrhea and chlamydia outcomes, the upper bound of the confidence interval was 1.01, suggesting that that our intervention likely had at least some small population-level effect. Of course, it is possible that our intervention was truly ineffective and that the decline in chlamydia test positivity and gonorrhea incidence observed during the study period was a consequence of a secular trend that was independent of our study. Rates of reported gonorrhea among women declined by 28% in California and 17% in Oregon during the study period, suggesting that a regional decrease in gonococcal incidence was occurring, though these declines were much smaller than the 53% decline observed in our study communities.

Several factors may have diminished our intervention’s impact, thereby decreasing our study’s statistical power. First, the intervention might not have reached a high enough proportion of people with gonorrhea or chlamydia, or might not have reached the persons most important in STI transmission. Second, the intervention’s effect may have been attenuated as a result of the non-independence of STI transmission between LHJs. Insofar as residents of study communities had sex with partners from LHJs other than their own or obtained care in LHJs outside their own, interventions in early wave communities could have lowered rates of STIs in other areas, leading to a general decline in gonorrhea and chlamydial infection that was not in step with our community randomization sequence. We do not have data on what proportion of partnerships involve persons in different LHJs. Third, our intervention could have leaked into areas that were still awaiting its institution, thereby diminishing our intervention effect. We observed relatively high levels of PDPT use at baseline in wave 4 (22%), but there was no clear trend toward increasing use of PDPT outside of the trial, as evidenced by the low level of PDPT use at baseline in wave 3 LHJs (10%). Fourth, the state’s unanticipated increase in the provision of partner services outside of the study may have contributed to observed decreases in STIs. Because this increase in the provision of partner services did not occur in synchrony with the study’s randomization sequence, it may have diminished our study’s power to see an effect and the intervention’s effect size. Lastly, the fact that only 23 LHJs participated in the trial (compared to 24 in the design) and that the effective average (harmonic mean) sample size per cluster per time period was 107 (compared to 162 in the design) resulted in a modest reduction in power relative to that anticipated in our study design (67% versus 80%).

The relatively small population-level effect we observed is consistent with findings from a recent mathematical model that suggested that increasing partner treatment may have only a small impact on chlamydia and gonorrhea prevalence [27]. However, prior models have suggested the opposite conclusion [28,29], and no gonorrhea or chlamydia model has been validated, making the predictive value of these models’ findings uncertain.

Our study demonstrates that a public health program that provides widespread access to free PDPT can lead to very large increases in PDPT use. The CDC and numerous US states currently have guidelines that include recommendations on the use of PDPT for gonorrhea and chlamydial infection [19,30–35]. The extent to which the intervention is used is not precisely known. A recent CDC study reported that only approximately 9.5% of persons diagnosed with gonorrhea in the US in 2010 received PDPT [11]. That study included MSM, a population in which PDPT is used less frequently and that we did not include in our study, and excluded from analysis persons whose partners were treated in the absence of PDPT, inflating estimates of PDPT use by an amount that is unknown. Despite these differences in study design, the use of PDPT for gonorrhea in WA State during our intervention period was clearly very much higher than that observed in any other area of the US for which data are available. How often medical providers outside of WA State use PDPT for chlamydial infection is not well defined. Studies surveying providers in the US have typically found that half or more report ever using PDPT, with 15%-50% reporting that they use it routinely, usually, or always [8–10]. However, the validity of these surveys is uncertain. To our knowledge, only one study including patients from more than a single clinic has evaluated PDPT use among persons with chlamydial infection outside of WA State; it found that 19% of 743 women diagnosed with chlamydial infection in nine California family planning clinics in 2005–2006 received PDPT [13]. This number is somewhat lower than the baseline level of 29% PDPT use by WA State family planning providers in our pre-intervention period, and well below the 53.5% observed following implementation of our study intervention.

We believe that our findings should promote increased efforts to expand the use of EPT. The effectiveness of EPT is supported by randomized trials demonstrating an individual-level benefit [3–6] and by a cost-effectiveness analysis [36]. Our study showed that a public health intervention could lead to a very large increase in PDPT use and, although not definitive, suggests that the increased use of EPT resulted in a modest decrease in population-level measures of gonorrhea and chlamydial infection in women. The goal of the trial, to definitively impact population-level measures of STIs over a relatively short period of time, was ambitious. Few data exist from community-level randomized trials, and the study designs are themselves experimental. Although several community-level randomized trials have evaluated interventions to prevent STIs in lower income nations [37]—some of which observed population level effects [38–41]—to date, only one such trial has been completed in a high-income nation [42]. That study, which was conducted in the Netherlands to evaluate the effect of chlamydial screening, was not successful in increasing chlamydial screening (the study intervention) and did not demonstrate an effect on STI prevalence. There are no community-level trials demonstrating the population-level benefits of STI clinical care, STI screening, traditional partner services, or the promotion of condom use, all of which are mainstays of STI control in high-income nations. Thus, while community-level randomized trials are a good aspirational evidentiary standard and additional trials of this type should ideally be undertaken, they are not currently a practical standard for defining which STI prevention interventions should be adopted.

The role of EPT in the control of gonorrhea merits specific comment. Current STD treatment guidelines in both the US and Europe do not recommend the routine use of cefixime as a therapy for gonorrhea [43,44]. At the same time, US guidelines continue to recommend the use of cefixime and azithromycin as an EPT regimen and as an alternative therapy for gonorrhea [43,45]. *N. gonorrhoeae* with diminished susceptibility to cefixime is uncommon in the US, causing fewer than 0.5% of all gonococcal infections in heterosexual individuals in 2013 (R. Kirkcaldy, personal communication). In Seattle, as of July 2014, we have not identified any *N. gonorrhoeae* isolates with decreased susceptibility to cefixime for more than 2 y. While additional research on this issue is needed, simple mathematical models suggest that eliminating the use of EPT could increase the transmission of gonorrhea overall, and under some instances might increase the transmission of gonococci with diminished susceptibility to oral third-generation cephalosporins [46]. Randomized trials have found that EPT decreases the absolute risk of gonococcal reinfection by 9% or more [4,5]. Given this very large effect size, the current rarity of gonococci with diminished susceptibility to cefixime among heterosexual individuals in the US, and the potential deleterious effect of eliminating EPT on gonococcal transmission, we believe that, at least at present, public health authorities in the US should continue to promote the use of EPT.

Our chlamydial outcome measure was based on test positivity in a sentinel population, and trends in that population may not have been representative of trends among all women in WA State. Women in IPP clinics were younger than the population of sexually active women ages 14–25 y in WA State overall, and it seems likely that the population in which we measured outcomes was at higher risk for STIs than the total population of women in the state. Also, the composition of the IPP population may have changed over time in ways that could have influenced the study outcome. Our analysis included random effects for LHJs and for clinics within LHJs to adjust for changes in the study population, but this adjustment may not have completely eliminated confounding. Ideally, our trial would have measured chlamydial prevalence in a random sample of women in each LHJ. However, such a design was not feasible given the resources available for the study.

Our measures of PDPT use by medical providers were based on surveys conducted with randomly selected patients being treated for gonorrhea or chlamydia, not all of whom were successfully interviewed. Although we weighted our estimates to adjust for nonresponse, our estimates could still be affected by bias if factors other than those used to weight the data affected our response rate. While this limitation affects the precision of our estimates of PDPT use, the use of PDPT in WA State during the study period was several times higher than that observed in the US as a whole [11], strongly suggesting that our intervention had a large population-level effect on PDPT use.

In conclusion, we found that a public health program that promoted use of free PDPT substantially increased clinicians’ PDPT use. Although our trial did not definitively demonstrate that EPT decreases chlamydia test positivity or gonorrhea incidence, we did observe substantial population-level declines in both infections concurrent with the introduction of our intervention. WA State has continued to make free PDPT packs available statewide over the 3 y since our study ended, and the number of packs distributed in the state has remained relatively stable in the post-study period, demonstrating that a state-wide system of EPT provision is sustainable. To our knowledge, no other US state or area outside of the US has developed a system similar to WA State’s. Given existing evidence and guidelines in support of EPT [3–5,23], we believe that continued and expanded use of the intervention is warranted, and that the design of our program can be a model for health departments seeking to increase EPT use.

## Supporting Information

S1 Text Study protocol. (PDF)

S2 Text Analysis plan. (DOCX)

S3 Text Ethics review documentation. (PDF)

S4 Text CONSORT checklist. (DOC)

## Abbreviations

CDC: United States Centers for Disease Control and Prevention
EPT: expedited partner therapy
IPP: Infertility Prevention Project
LHJ: local health jurisdiction
MSM: men who have sex with men
PDPT: patient-delivered partner therapy
STI: sexually transmitted infection
WA: Washington
