# Supplementary material for: Uptake and Population-Level Impact of Expedited Partner Therapy (EPT) on Chlamydia trachomatis and Neisseria gonorrhoeae: The Washington State Community-Level Randomized Trial of EPT
Source: PLoS Med. 2015 Jan 15;12(1):e1001777. doi: 10.1371/journal.pmed.1001777 (PMC4295847; doi:10.1371/journal.pmed.1001777)
Supplement: S1 Text — (PDF) [file pmed.1001777.s001.pdf]

Protocol
Washington State Expedited Partner Therapy Program for Gonorrhea and Chlamydial Infection

Updated April 2011

Table of Contents

	Pages
I. Overview	3-6
II. Case report forms and data management	7-16
III. Partner Notification Interview procedures*	17-18
IV. Expedited Partner Therapy	19-21
V. Pharmacy Protocol	22-24
VI. Sample Clinic Protocols	25-28

***See** Expedited Partner Therapy SSuN Case Interview Protocol for information about specific interview questions

OVERVIEW

Background and Rationale

Partner notification and partner treatment are longstanding cornerstones of public health efforts to control sexually transmitted infections (STIs), including gonorrhea and chlamydial infection. Traditionally, health department efforts designed to assure partner notification and treatment have involved attempts to interview infected persons (index patients), elicit contact information about their sex partners, and assure their partners were treatment. This approach was developed to control syphilis in the period around World War II, and was subsequently applied to other STIs. However, most health departments do not have resources to interview all persons with bacterial STIs, and in recent years only a minority of persons with gonorrhea or chlamydial infection nationally, and in Washington State, have been contacted by public health authorities[1]. Recognition of the inadequacies in the traditional U.S. approach to partner notification led the Institute of Medicine to advocate a redesign of public health partner notification programs in 1997[2].

Expedited partner therapy (EPT) is an alternative to traditional partner notification. EPT refers to the practice of treating the sex partners of persons with STIs without requiring that they first see a medical provider. In practice, EPT usually involves patient-delivered partner therapy, or PDPT (i.e. giving an index patient medication to give to their sex partners). Observational studies conducted in the 1990s, as well as three recent randomized controlled trials, have found that providing persons with gonorrhea or chlamydial infection with decreases rates of reinfection and increases the proportion of potentially exposed sex partners who are treated [3-7].

In response to mounting evidence in support of PDPT, the Washington State Department of Health (DOH) issued guidelines on the use of PDPT in 2004. These guidelines, subsequently unanimously endorsed by the Washington State Health Officers, suggest that providers offer PDPT to all patients they diagnose with gonorrhea or chlamydial infection if they are not sure that the patient's potentially exposed partners will be evaluated and treated as indicated.

Starting in 2004, Public Health – Seattle & King County (PHSKC) initiated a CDC funded, county-wide EPT program that provided access to free PDPT packages to all clinical providers in the county, and used revised case report forms to triage index patients at high risk for partner notification failure to the health department for partner notification assistance. An evaluation of that program found that 81% of providers completed the partner notification of the case report form, and that persons referred to the health department for assistance were significantly more likely to have untreated partners and more likely to accept assistance notifying their partners. PHSKC investigators estimated that the program increased the proportion of index cases all of whose partners were treated from 39% to 61% concurrent with the new program.

A Statewide EPT Program
Based on evidence supporting the efficacy of EPT and encouraging experience in King County, in 2005 University of Washington (UW) scientists and public health officials at the Washington State DOH developed a proposal to the National Institutes of Health (NIH) to fund a state-wide EPT program. That proposal was funded in 2006 and pays for EPT to be instituted state-wide as part of a research study. The study pays for staff to assist instituting the intervention (including new Disease Intervention Specialists [DIS], medication and pharmacy fees association with EPT, and development and management costs for a web-based case reporting and partner notification system. The partner notification procedures instituted as part of the statewide program are not experimental since they conform to existing Washington State DOH guidelines and established PHSKC routines. However, the program will be instituted in different local health jurisdictions in a randomly selected order, which is a research design.

The basic components of the new system include:

1) An Internet-based case report and partner notification data management system;
2) Standardized partner notification interview records – these records were developed both for this project and for another CDC funded project, SSUN;
3) New case report forms that ask providers to indicate whether they want to manage an index case's partners or have the health department manage partners. This form also includes a pre-printed prescription that can be faxed to pharmacies to allow index patients or partners to obtain partner packs;
4) Access to free medications for EPT for clinical providers throughout the state
   – Medications are packaged in "partner packs" to meet state pharmacy board requirements
   – Partner packs will be distributed directly to clinics and provider offices that report large numbers of gonorrhea or chlamydia cases
   – Selected commercial pharmacies in each jurisdiction will stock partner packs to distribute to index patients and their partners.

Each of these components is discussed in greater length in the subsequent sections of this protocol.

Program evaluation
The program evaluation will include an assessment of process outcomes as well as community-level monitoring of the prevalence of infection. Program staff will assess what proportion of persons diagnosed with gonorrhea or chlamydial infection receive PDPT and report that their partner(s) were treated. To accomplish this, a random sample of reported cases will be called and interviewed. These interviews will be conducted in collaboration with local health jurisdictions under existing public health authority to conduct investigations into cases of STI.

The prevalence of infection will be monitored in Infertility Prevention Project (IPP) clinics, and incidence will be estimated using case reporting. IPP clinics employ standardized criteria for performing tests for gonorrhea and chlamydial infection. Because the order of program institution will be random, changes in the prevalence and incidence of infection can be compared in areas where PDPT is being implemented in early phases to those areas joining the implementation in later phases.

Benefits to participating counties

The anticipated public health benefit of participating in this PDPT initiative will be measurable reductions in the incidences of gonorrhea and chlamydial infection statewide, and heightened awareness within the provider community of the importance of partner treatment for preventing reinfection.

Participating counties will receive assistance implementing the PDPT program, access to a new data management system, and pre-packaged medications for PDPT at no cost. Some counties that currently have no Disease Intervention Specialists (DIS) also will receive DIS support. Participating jurisdictions will also receive epidemiologic support to evaluate the PDPT program locally. Program implementation will be coordinated with public health officials in each local health jurisdiction as well as with the state STD/TB Services Section. Local jurisdictions with DIS available will be able to continue selectively referring patients for DIS intervention based on the presence of absence of specific risk criteria.

Local public health responsibility

Each local jurisdiction will be responsible for identifying an official to coordinate their activities with the State Department of Health and University program staff. Jurisdictions joining the project will also be required to limit local PDPT promotion to the randomly assigned period (i.e. areas assigned to initiate the program later will not be able to undertake separate PDPT promotion programs prior to the state program). To the extent that local jurisdictions currently provide any partner notification services, it is expected that those services would be integrated into the new system, including use of PDPT, a standardized interview record, and a web-based case management system. The standardized interview record is being promoted throughout the state independent of this project.

Program oversight

Faculty from the University of Washington's Center for AIDS and STDs and personnel from the Washington State Department of Health, STD/TB Services Section will collaboratively direct program activities. Dr. Matthew Golden will provide overall leadership in grant development, and program implementation and evaluation for the University of Washington. Mark Stenger, STD/HIV Epidemiologist, will serve as the State Department of Health's program coordinator. Health Officers in participating jurisdictions will establish an advisory committee to monitor the program's implementation and evaluation and to provide feedback to the program directors.

Sustainability
A major long-term goal of this project will be to help local jurisdictions develop the resources to sustain PDPT program activities. In collaboration with an economist at CDC, program staff will monitor the costs associated with ongoing activities to establish what resources will be needed to sustain widespread promotion of PDPT beyond the grant funded period. In addition, the state will actively pursue federal funding for PDPT should resources become available. However, participation in this project carries no guarantee of ongoing funding.

References:

1. Golden MR, Hogben M, Handsfield HH, St Lawrence JS, Potterat JJ, Holmes KK. Partner notification for HIV and STD in the United States: low coverage for gonorrhea, chlamydial infection, and HIV. Sex Transm Dis 2003; 30:490-6.
2. Institute of Medicine (U.S.). Committee on Prevention and Control of Sexually Transmitted Diseases., Eng TR, Butler WT. The hidden epidemic : confronting sexually transmitted diseases. Washington, D.C.: National Academy Press, 1997:xii, 432.
3. Ramstedt K, Forssman L, Johannisson G. Contact tracing in the control of genital Chlamydia trachomatis infection. Int J STD AIDS 1991; 2:116-8.
4. Kissinger P, Brown R, Reed K, et al. Effectiveness of patient delivered partner medication for preventing recurrent Chlamydia trachomatis. Sex Transm Infect 1998; 74:331-3.
5. Schillinger JA, Kissinger P, Calvet H, et al. Patient-delivered partner treatment with azithromycin to prevent repeated Chlamydia trachomatis infection among women: a randomized, controlled trial. Sex Transm Dis 2003; 30:49-56.
6. Golden MR, Whittington WL, Handsfield HH, et al. Effect of expedited treatment of sex partners on recurrent or persistent gonorrhea or chlamydial infection. N Engl J Med 2005; 352:676-85.
7. Kissinger P, Mohammed H, Richardson-Alston G, et al. Patient-delivered partner treatment for male urethritis: a randomized, controlled trial. Clin Infect Dis 2005; 41:623-9.

II) CASE REPORTS

New Case Report Forms

Part of the EPT program is use of a new case report form. There are two major differences between this form and the traditional WA state case report: 1) The case report asks providers to indicate how they want to manage their patient's sex partners; and 2) the form includes a preprinted prescription for medication to use for EPT. These changes are designed to convert partner notification from a passive to an active process for providers, forcing them to commit to a partner notification plan. It is also designed to give them new tools to help get their patient's partners treated.

<u>Defining a partner notification plan</u>

The case report includes two shaded sections related to partner management. The 2nd page of the report has a shaded section entitled, "Instructions for Partner Management Plan." This section advises provider to complete the section on the 3rd page, and lets them know that free medication is available for PDPT and how to obtain it. The 3rd page of the case report includes the section "Partner Management Plan" (see below). Providers should complete this section of the case report indicating one of 3 options:

1) Health department to assume responsibility for partner treatment – persons for whom this box is checked should be contacted by public health staff for partner notification interview.
   - This option is recommended for persons with any of the following risks:
     - >1 sex partner in the preceding 60 days
     - Patient reports a having a sex partner s/he does not think they will have sex with again
     - Patient is unwilling or unable to contact a partner
     - Patient is a man who has sex with men
2) Provider will ensure all partners are treated
   - In most instances, these patients should be offered PDPT
   - Providers are asked to indicate how many partners they treat
3) All partners are already treated
   - Providers are asked to indicate how many partners they treat

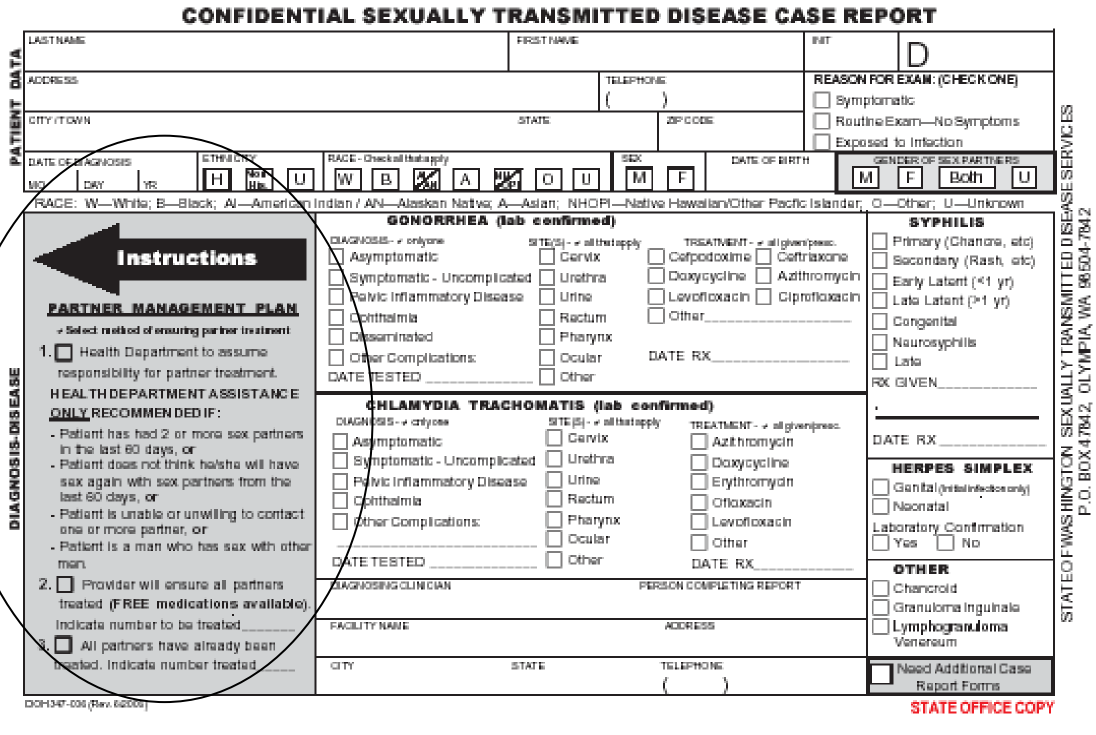

CONFIDENTIAL SEXUALLY TRANSMITTED DISEASE CASE REPORT

PATIENT DATA

LAST NAME | FIRST NAME | INIT | D

ADDRESS | TELEPHONE ( )

CITY / TOWN | STATE | ZIP CODE

REASON FOR EXAM: (CHECK ONE)
☐ Symptomatic
☐ Routine Exam—No Symptoms
☐ Exposed to Infection

DATE OF DIAGNOSIS: MO DAY YR | ETHNICITY: H Non His. U | RACE - Check all that apply: W B AI/AN A NH/OPI O U | SEX: M F | DATE OF BIRTH | GENDER OF SEX PARTNERS: M F Both U

RACE: W—White; B—Black; AI—American Indian / AN—Alaskan Native; A—Asian; NHOPI—Native Hawaiian/Other Pacific Islander; O—Other; U—Unknown

DIAGNOSIS-DISEASE

Instructions

PARTNER MANAGEMENT PLAN
✓ Select method of ensuring partner treatment
1. ☐ Health Department to assume responsibility for partner treatment.
HEALTH DEPARTMENT ASSISTANCE <u>ONLY</u> RECOMMENDED IF:
- Patient has had 2 or more sex partners in the last 60 days, or
- Patient does not think he/she will have sex again with sex partners from the last 60 days, or
- Patient is unable or unwilling to contact one or more partner, or
- Patient is a man who has sex with other men.
2. ☐ Provider will ensure all partners treated (FREE medications available). Indicate number to be treated_______
3. ☐ All partners have already been treated. Indicate number treated _____

GONORRHEA (lab confirmed)
DIAGNOSIS - ✓ only one: ☐ Asymptomatic ☐ Symptomatic - Uncomplicated ☐ Pelvic Inflammatory Disease ☐ Ophthalmia ☐ Disseminated ☐ Other Complications:
DATE TESTED ___________
SITE(S) - ✓ all that apply: ☐ Cervix ☐ Urethra ☐ Urine ☐ Rectum ☐ Pharynx ☐ Ocular ☐ Other
TREATMENT - ✓ all given/presc.: ☐ Cefpodoxime ☐ Ceftriaxone ☐ Doxycycline ☐ Azithromycin ☐ Levofloxacin ☐ Ciprofloxacin ☐ Other_______________
DATE RX_______________

CHLAMYDIA TRACHOMATIS (lab confirmed)
DIAGNOSIS - ✓ only one: ☐ Asymptomatic ☐ Symptomatic - Uncomplicated ☐ Pelvic Inflammatory Disease ☐ Ophthalmia ☐ Other Complications: ____________________
DATE TESTED ___________
SITE(S) - ✓ all that apply: ☐ Cervix ☐ Urethra ☐ Urine ☐ Rectum ☐ Pharynx ☐ Ocular ☐ Other
TREATMENT - ✓ all given/presc.: ☐ Azithromycin ☐ Doxycycline ☐ Erythromycin ☐ Ofloxacin ☐ Levofloxacin ☐ Other
DATE RX___________

SYPHILIS
☐ Primary (Chancre, etc) ☐ Secondary (Rash, etc) ☐ Early Latent (<1 yr) ☐ Late Latent (>1 yr) ☐ Congenital ☐ Neurosyphilis ☐ Late
RX GIVEN___________
DATE RX ___________

HERPES SIMPLEX
☐ Genital (initial infection only) ☐ Neonatal
Laboratory Confirmation ☐ Yes ☐ No

OTHER
☐ Chancroid ☐ Granuloma Inguinale ☐ Lymphogranuloma Venereum

DIAGNOSING CLINICIAN | PERSON COMPLETING REPORT

FACILITY NAME | ADDRESS

CITY | STATE | TELEPHONE ( )

☐ Need Additional Case Report Forms

STATE OF WASHINGTON SEXUALLY TRANSMITTED DISEASE SERVICES
P.O. BOX 47842, OLYMPIA, WA 98504-7842

DOH 347-036 (Rev. 6/2006)

STATE OFFICE COPY

Prescriptions

The last page of the case report is a preprinted prescription for public health treatment packs. Providers can complete these forms and fax them to pharmacies participating in the program, allowing their patients to receive free medication for their sex partner(s). The forms must be completed, including a name of the intended recipient of the medication (i.e. the partner to be treated) and the name of the person picking the medication up (i.e. usually the index case). Although the health department pays for these medications, when sent to pharmacies by a provider, the provider is the prescribing clinician and must sign the form.

WASHINGTON STATE EXPEDITED PARTNER THERAPY PROGRAM

PHARMACY: ______________________ DATE: ____________

TELEPHONE: ______________________ FAX: ____________

Rx: PATIENT NAME: ______________________ DOB: ____________
(INTENDED RECIPIENT)

PERSON PICKING UP MEDS: ______________________ DOB ____________

Rx: DISPENSE MEDICATIONS AS CHECKED BELOW AT
NO CHARGE TO PATIENT.

MEDICATIONS TO BE DISPENSED WITHOUT CHILDPROOF SAFETY CAP.

☐ PUBLIC HEALTH - PACK 1 – AZITHROMYCIN, 1 GRAM (ZITHROMAX)
po once stat

☐ PUBLIC HEALTH – PACK 2 – AZITHROMYCIN, 1 GRAM (ZITHROMAX) po stat to be dissolved in water or other drink, CEFPODOXIME 200 MG (VANTIN) 2 tablets (400mg) once po stat.

X Dispense as written

____
PHYSICIAN SIGNATURE

Data management of case reports

1. Case Report Flow
   a. Case reports come to local health departments/LHJs via the USPS mail/FAX on a daily basis. All reports need to be opened in a timely manner for data entry/case management at the health department/LHJ office site or for faxing to DOH/UW staff in Seattle for data entry/case management.
      i. If DOH/UW staff are handling data entry/case management for an LHJ, case reports need to be faxed on a daily basis.
      ii. The confidential fax number for which to send reports is: **206-744-5622**

b. Look at the address of the patient in the Patient Data section on the case report form. If the pt does not live in your LHJ (see zip code listing), this case is Out of Jurisdiction or OOJ.
    i. Follow the transfer guidelines detailed in **Transferring a Case**
    ii. If the patient pt does not reside in Washington State the entire case report needs to be forwarded onto the Washington State Department of Health (DOH) in Olympia.
        1. OOJ case reports need to be mailed with the daily mailing of completed reports - see below.

c. Each case report needs to be reviewed carefully to ensure that the following has been done:
    i. The form is completely filled out based on the diagnosis. For incomplete forms:
        1. Fax the case report along with the Case Report Fax Cover Sheet to the provider requesting the needed information.
        2. Make two faxing attempts one week apart.
            a. If no response, make one phone call to obtain the needed information. No more attempts should be made after the phone call. Consider the case report final at this point.
    ii. Ensure the treatment listed on the report is adequate for treating the designated infection (see **STD Guidelines Section**.) If a patient is not adequately treated, the case needs to be referred to a DIS or UW program staff for follow-up and treatment.
        1. Examine the date of birth - if patient is 13 years of age or younger, follow your LHJ guidelines for sexually active patients under 14 years of age.

d. Enter the case report into the STIMS database. Please see additional explanation for this task in the **Data Entry Section**

2. Data Entry Procedures

a. Background
    i. One of the most important tasks of data entry is to ensure that we have an accurate and up-to-date case registry. To ensure an accurate registry, several things need to happen:
        1. First, there is an expectation that labs and case reports get entered into the database in ***a timely manner meaning within 48 hours of receiving them*** at the most. That said, if you have been sick or on vacation, it is understood it may take additional time to get caught up.
        2. Second, it is very important that the database be populated with accurate case data. Please beware of duplicate cases, misspellings, typos etc. PHIMS-STD is a medical record like the records you have on file at your doctors' office. We cannot tolerate errors to the electronic records stored in PHIMS-STD.

a. Whenever you are planning to enter a new case/positive result into the computer it is ***absolutely imperative*** that you look by both date of birth and name to see if the patient is already in the database. In addition, you want to ensure that you are not about to enter case data for a positive result that is already in the STIMS system.

3. The assignment of cases is to DIS and UW program staff is determined by sampling cases during the data entry process. It is absolutely imperative that with every case that is entered into PHIMS-STD that the assign sample button be clicked at the bottom of the case report page.

b. Case Reports

i. Case reports come to the Health Department/LHJ via the FAX/USPS mail. Remember to check the name and date of birth on the case report against cases in PHIMS-STD.

ii. The usual scenario is that labs come prior to case reports. Obviously, this means that the case should be in the PHIMS-STD already. It is very important for you to check the database to ensure you do not create a duplicate case record for the same disease event.

iii. If the patient has had a previous STD case, but does not have a case for the date of service listed on the case report in front of you, you can populate a new case form in STIMS with some existing information on this patient by selecting **NEW** at the far right of the patients previous case information

1. If the patient has multiple previous positives it is best to select the most recent case as address and phone # information is automatically populated. The newer the information, the more likely it is to help in working the case.
2. Also, document in the notes section of the case report page, the month and years of previous positives. If they have 4 or more previous positives, note the total number of previous cases and not the actual dates.

iv. Case reports **must** be entered into PHIMS-STD exactly as they appear on the original paper form.

1. The only exception to this is if you have clarification from the providers' office that they have misspelled a patients' name or mistaken their date of birth.
2. in this instance you want to correct the paper form as well as correct the electronic data in PHIMS-STD

v. From time to time, we get more than one case report for the same disease.

1. Examine the reports to ensure they are completed with identical information.

a. If one report has updated information or is more complete, update the electronic record in PHIMS-STD notating in the notes section of the database that a

duplicate case report was received along with the new case report #.

vi. If you have an incomplete case report, please follow the faxing procedures outlined in the **Case Report Flow** section above

vii. Case reports are in part what we use to assign out cases for the DIS and Partner Management staff to work. This will be detailed more in the **Case Assignment** section**.**

c. Lab Reports

i. In some counties/LHJs, there may be an arrangement with the local labs concerning the reporting of STDs directly to the Health Department/LHJ via mail or fax.

ii. It is not uncommon to get several copies of the same lab result.

1. Often times the duplicates have additional or updated information.
2. If you think you have seen the lab report before, it is best to give it the once over carefully to make sure that you are not missing any updated or additional information.

iii. Remember to check the name and date of birth on the lab against cases in PHIMS-STD.

iv. Procedures for entering labs are identical to the procedures for case report data entry listed in **Case Report Flow** above

v. Remember to check the name and date of birth on the lab against cases in PHIMS-STD.

vi. Lab reports do come to us on a regular basis prior to us receiving the case report. It is necessary to initiate a case in the PHIMS-STD system before we get the actual paper case report form. This instance is the only time that you would populate any field on the Case Report page in PHIMS-STD without having the actual paper case report form! There is no exception to this rule.

1. Limited information is input into the Case Report page from the lab report
   a. name
   b. date of birth
   c. disease
   d. site
   e. date tested

vii. Lab tracking information also needs to be entered into PHIMS-STD. This information gets put into the database on the very bottom portion of the Case Report page. The fields to be populated include:

1. date of initial lab report - the date we received the lab report
2. lab name - the lab that processed and reported the positive result
3. provider - name of doctor (in some case clinics) who collected specimen
4. provider phone # - phone # of provider (clinic)

5. referral basis - that is the way in which we first hear about the positive case

d. Providers/Clinics
   i. Each case in the new system needs to have a provider assigned. This is in most cases a doctor, nurse practitioner, or physician's assistant.
   ii. In all cases possible, there also needs to been a clinic or facility assigned. This feature allows us the ability to account for providers who work in multiple facilities.
      1. There are again a few exceptions to this rule.
   iii. Be sure to verify what you have typed if you are updating or adding a new provider.. Many mistakes in provider lists are the result of something as small as a minor typo. In addition, mistakes can be prevented by taking a few minutes to verify the information we have with a receptionist when we are calling regarding another matter related to the case.

   New clinics or change in clinic information should be emailed to the DOH Julieann Simon Julieann.Simon@DOH.WA.GOV so the PHIMS-STD system can be updated.

3. Duplicates, False Positives, Equivocals

a. In PHIMS-STD, all duplicate cases and false positives need to be entered. The reason that we record duplicate cases and false positives is to reconcile people who frequent multiple facilities within a short period of time and to have record of cases that have been deemed a false result.

b. Duplicates
   i. A duplicate positive is a case in which a patient tests positive twice within a 3 week period regardless of whether the patient has received treatment. The two cases should be considered one event.
   ii. The two positives could be from the same provider/clinic or from two different providers/clinics
   iii. Both events must be entered into the PHIMS-STD system
      1. The second event (with the later test date) should be considered the duplicate and should be marked as so on the Case Report page in PHIMS-STD under the *Case Status* drop down menu
      2. Please make a remark in the notes section on the Case Report page in PHIMS-STD **on both cases** that there is a duplicate case, including the PHIMS-STD ID # and/or the case report # (if you have it) for reference
   iv. If there is a reported treatment for one case, ensure that it is marked on both case reports, in PHIMS-STD in both cases, and make sure both providers are aware of the disease treatment if the positive results came from two separate facilities
   v. Only one of the two cases should be assigned out and advise the DIS or UW staff person working the case of the duplicate positive

c. False Positives
    i. A false positive result is one in which the original test result is inaccurate. These are reasonably rare though they do happen. Some examples are:
        1. There is a specimen contamination at the lab
        2. An incorrect result(s) is faxed due to a recording error at the lab
        3. Using tests that are not recommended screening tools for a given bacteria

d. Equivocals
    i. We sometimes get labs and case reports for indeterminate test results. We consider these equivocals and we do not record these results.
    ii. If you get a case report or a lab on an equivocal result please follow these procedures:
        1. Notify the diagnosing provider that this is not considered a positive case and that these do not need to be reported
            a. Ensure that the patient is the process of being retested or has been so already

4. Transferring a Case/Out of Jurisdiction (OOJ)

a. An OOJ case is:
    i. A case that was test in another county but is referred to your county/LHJ for treatment and/or follow-up as it is the patients county of residence
    ii. A case that was tested in your LHJ but who's residence is in another state or county
    iii. OOJs are generally referred through Julieann Simon at the DOH in Olympia.

b. OOJ cases that are referred to your Health Department/LHJ for follow-up
    i. A patient that was tested in another county/LHJ/state that is not treated and does not live in the jurisdiction in which they were tested. It falls on your jurisdiction to ensure this patient is treated for the positive infection
        1. There are several ways you may here about an OOJ case that is referred to you LHJ
            a. A field record will arrive from the DOH in Olympia.
            b. A phone call may come to you from a DIS in another LHJ
            c. A new case in PHIMS-STD may appear as unassigned and have an OOJ notification message in the Notes section on the Case Report page
        2. If the case comes via a field record or by phone, follow the instructions for entering a new case into the database listed in the Data Entry Section.
        3. You may have very limited information on the case from the field record or phone call. Please enter any information you receive with the understanding that some of it may need to be typed into the notes section.

4. Once the case is in PHIMS-STD, it should be assigned out to the appropriate worker for follow-up and/or treatment
5. A case report may eventually float in from the DOH in addition to the field record you may have already received.

c. OOJs that need to be referred from your LHJ to another jurisdiction
    i. A patient who tests positive in your LHJ but whose residence resides outside of your LHJ.
        1. These cases need to be handled in one of two ways depending upon the receiving county
            a. The OOJ cases will can transferred electronically through PHIMS-STD
                i. Upon receiving notification of the positive and verifying address information for the patient, complete the case information on the Case Report page in PHIMS-STD
                ii. At the bottom of the Case Report page, click the **Transfer** button. A screen will appear asking you to choose and verify the sending and receiving counties. When you have confirmed that the sending and receiving counties are correct, continue in transferring the case.

5. Contact To Cases

a. A "contact to" is a named partner to a known STD case. It is someone who has been exposed to an STD.
b. Contact to cases can be, like an OOJ, referred both to and out of our jurisdiction.
c. Contact to cases should be handled like cases and OOJs.
    i. Cases may get referred to us by field record, by phone from Julieann Simon at the state, or electronically through STIMS
d. If a contact to case is referred to you from another county via phone or field record, this should be entered into PHIMS-STD and assigned out to an appropriate worker.
    i. As above, remember that you may have very little information on this person - maybe only a name and phone number.
    ii. Still enter this into the system make a note in the case report notes section in PHIMS-STD that it is a contact to.
    iii. On the lab section on the Case Report page, under the referral basis drop-down, please select the "Ptx Referred Partner" option.
e. If a contact to case is referred to your LHJ electronically through PHIMS-STD, it will appear as an unassigned case with a note in the notes section on the Case Report page that the case is a contact to
f. Contact to cases that are referred out of our jurisdiction
g. DIS will be primarily responsible for this as they handle their individual cases.

6. Case Assignment/Sampling

a. Cases need to be assigned out based on several important criteria that are defined as follows:
    i. A specific case is part of the fixed sample
    ii. A specific case is part of the variable sample
    iii. An incoming case report is marked as requesting public health assistance with partner notification and treatment

b. Fixed Sample
    i. 20% of the total number of heterosexual CT, GC and CT/GC cases need to assigned out to a DIS in your LHJ or to a UW staff person working on the study for contact for interview
    ii. Upon creation of a case in PHIMS-STD, the assign sample box must be clicked at the bottom right hand corner of the Case Report page
    iii. "Y' means the case is a part of the fixed sample and must be assigned out for interview
    iv. "N" means the case is not part of the fixed sample.
    v. Cases may be closed when a case report has been received and a treatment appropriate for the case's diagnosis has been marked.
    vi. If the "Health Department to assume responsibility for partner treatment" box is marked, this case needs to be assigned out for interview:
        1. Even if it is not part of the fixed sample
        2. And only if the case report has been received within 30 days of the specimen date

c. Variable sample
    i. For those counties with the capability to contact more than the required 20% of cases in the fixed, an additional percentage of cases will be sampled for interview
    ii. This additional percentage is to be determined by the LHJ
    iii. "Y' means the case is a part of the variable sample and must be assigned out for interview
    iv. "N" means the case is not part of the variable sample.
    v. If the "Health Department to assume responsibility for partner treatment" box is marked, this case needs to be assigned out for interview:
        1. Even if it is not part of the variable sample
        2. And only if the case report has been received within 30 days of the specimen date

d. Requests for Health Department Assistance
    i. The new case reports ask providers to select a partner management plan for their patient's sex partners. Specifically they must indicate:
        1. If they would like the Health Department to assume responsibility for the treatment of their patient's partners
        2. That they have treated all of their patient's partners
        3. That all of their patient's partners have been treated

ii. All cases for which the “ Health Department to assume responsibility for partner treatment” box is marked must be assigned out to DIS or UW program staff for interview
    1. This holds true even if the cases is not sampled for interview in either the fixed or variable sample
    2. This holds true only when the case report arrives within 30 days of the specimen date listed on the case report form and/or lab report

III) Partner Notification Interviews

1) Contacting index cases
    a. DIS will initiate all efforts to contact index cases as soon as possible following case assignment
    b. DIS should make at least 5 attempts to contact OP by phone, mail, email or text.
        i. Telephone contact
            1. Confirm that the person to whom you are speaking is the OP using their date of birth and identify of recent medical provider
            2. Confirm that the OP knows their STD diagnosis, that they have been treated for their infection and they understand their diagnosis. Then ask them if their partner/ partners have been treated.
                a. If **not treated**: tell the patient that you can help them with getting their partner(s) treated for **FREE**. Tell them that you need to ask them some questions before you call in their partner's prescription. Tell them that they can refuse to answer any questions and that all their answers are confidential.
                b. If they **are treated**: ask them if they would like to help in reducing STD infection rates by answering some questions. All questions they answer are confidential and they can refuse to answer any question(s).
            3. Voice mail – DIS should initially leave only their first name on a message. After leaving several voicemails with no response, indicate that you are calling with about a health matter.
            4. Messages with persons other than the OP – DIS should leave their name only. If the note-taker ask want it is concerning, tell them you are following-up on an appointment that (name of index patient) had on MM/DD. If they are still persistent about where you are calling from tell them HIPPA laws do not allow you to give out that information.
        ii. Letters
            1. Letter should be sent in plain envelopes that do not mention PHSKC or STD
            2. Letters should indicate that the DIS needs to talk to the case about an important health matter
        ii. Emailing and Texting should sent in accordance to LHJ protocol.
    c. Attempts to contact OP should continue for up to 30 days after the treatment date. When all attempts have been exhausted case should be close out according to interview status.
2) Initial interview
    a. Interviews should follow procedures laid out in the **EPT & SSUN Interview Protocol**
3) Re-interviews
    a. Reinterviews should be routinely done on the following persons:

   i. Persons with >1 sex partner in the 60 days prior to STD testing
   ii. Persons who report that they do not think that they will have sex again with one of their partners
   iii. Persons who agree to pick-up medications for partners but do not do so.

b. Goals of reinterview
   i. Ascertain if all partners are notified and treated
   ii. Offer to contact any unnotified or untreated partners
   iii. Offer index patient medications for partners and strongly encourage them to obtain those medications

c. Three attempts should be made to continue reinterviewing index cases until all partners are known to be treated. If unable to reestablish contact with OP or they fell to comply after three attempts proceed with final disposition.

d. The final disposition portion of the partner interview record should be completed after the final reinterview.

IV) Expedited Partner Therapy (EPT)

Candidate Populations for EPT

All heterosexuals with gonorrhea or genital chlamydial infection should be offered EPT if a provider cannot otherwise be certain that their partners will be treated. In addition, public health staff providing partner notification services should offer all heterosexuals diagnosed with gonorrhea or chlamydial infection medications for patient-delivered partner therapy (PDPT). Men with nongonococcal urethritis and women with mucopurulent cervicitis should not be offered PDPT until or unless they are diagnosed with gonorrhea or chlamydial infection. Also, the Washington State EPT program does not recommend routinely offering men who have sex with men (MSM) PDPT because of evidence that approximately 5% of the sex partners of these men with have undiagnosed HIV. Local health departments within the state may elect to use PDPT in MSM in circumstances to be defined locally.

Packaging EPT

All EPT dispensed via the Washington State EPT program will be dispensed in prepackaged medication. Prepackages will be prepared by the PHSKC pharmacy, which will be responsible for sending medications to pharmacies and clinics around the state and for maintaining data on medication expiration dates. Prepackaged medications include condoms and information about the drugs and about chlamydia and, in some instances, gonorrhea. The information included meets the Washington State Pharmacy Board's requirement that medication be dispensed with instructions and a warning regarding drug allergies.

Venues Stocking EPT

Medications for EPT can be provided either directly by clinicians using medication packets stocked in clinics or doctors' offices, or through designated local pharmacies. In each jurisdiction, the EPT Program will work with local health departments to define clinics or doctor's offices and pharmacies to stock prepacked EPT. The program will assure that pharmacies maintain EPT stocks. Local health departments and program staff will work together to assure that clinics and offices stocking EPT have a consistent supply of medications.

Direct Provision of Expedited Partner Therapy by Public Health Staff, Including DIS

Index patients and partners contacted by public health staff for purposes of partner notification will be routinely offered EPT as described above (see Candidate Populations for EPT). The following describes procedures for dispensing EPT to index patients and partners.

The last part of each partner notification interview involves defining a partner notification and treatment plan for each untreated partner. All index patients should be offered assistance notifying partners, and all heterosexual patients should be offered PDPT. Defining a partner notification plan involves the following steps:

1) Define who will notify and assure the treatment of each untreated partner (question 22 on the partner management record)

a) If the index patient wants to assure the partners treatment (questions 23),
   i. Do they want medication for PDPT?
   ii. If yes, how do they want to obtain it, from a pharmacy, in a clinic or via the mail?
   iii. If an index patient wants to obtain PDPT from a pharmacy:
      1. Decide which pharmacy
      2. Complete a prescription form and fax it to a pharmacy
      3. A clinician with prescribing authority must sign this form within 7 days.

b) If the index patient wants public health to contact a partner:
   i. Collect contact information
   ii. Contact partner and offer them referral for complete medical evaluation and EPT
   iii. If the partner wants EPT
      1. **Ask about drug allergies**
         a. If partner has a drug allergy to azithromycin (zithromax), erythromycin, or clarithromycin (biaxin), they cannot be prescribed a partner pack and should be referred to see a clinician.
         b. If partner exposed to gonorrhea has a drug allergy to penicillin, amoxicillin, or any cephalosporin, they cannot receive partner packs for gonorrhea and should be referred to see a clinician.
         c. When in doubt about a drug allergy, contact a medical provider.
      1. Define how the partner wants to obtain medication, from a pharmacy, in a clinic or via the mail?
         a. If an partner wants to obtain EPT from a pharmacy:
            i. Decide which pharmacy
            ii. Complete a prescription form and fax it to a pharmacy
            iii. A clinician with prescribing authority must sign this form within 7 days.

2) Follow-up for all prescriptions for EPT– Follow-up has two components:
   a) Determining if prescribed medications (PDPT or medications prescribed directly to partners) have been obtained.
      i. Run the weekly Prescription Pick-up Confirmation report from STIMS that lists the open prescriptions at each of your local jurisdiction pharmacies
      ii. Once a week, fax the Prescription Pick-up Confirmation report to the individual pharmacies to obtain pick-up confirmation
         1. If there is no response from the pharmacy within two days, re-fax the Prescription Pick-up Confirmation report
   b. Medications that have been picked-up
      i. As the completed reports are returned from the pharmacies:

1. Record the pick-up information on the original prescription form
2. Enter the pick-up information for each prescription into STIMS.
3. For those prescriptions that are confirmed by the pharmacy to have been picked-up, file the completed pharmacy form

c. Medications that have not been picked-up:
    1. Make a follow-up call to the person responsible for picking-up the prescription (usually the index patient) reminding them to get their prescription(s)
    2. File the prescription form back with other pending prescription pick-ups
    3. After three non-pick-ups from the pharmacy, a prescription must be cancelled:
        a. Call the pharmacy to cancel the prescription
        b. Note on the prescription form in red ink and in the database that the prescription was cancelled and on what day
        c. File the prescription form with other completed prescription forms
        d. If an index patient does not pick-up their prescription and it is cancelled after three non-pick-ups, follow your local health jurisdiction rules for ensuring the treatment of an untreated case of CT and/or GC

ii. If a partners' prescription is not picked-up and is cancelled after three non-pick-ups, file the completed form with other completed prescriptions forms. No further follow-up is pursued

V) Pharmacy Protocol for Expedited Partner Treatment (EPT)

A) Background

a. Expedited Partner Therapy (EPT) is the practice of treating the sex partners of persons diagnosed with an STD without requiring that the partner first see a clinician.
b. In most instances, EPT involves giving a patient medication to give to their sex partner(s), something called patient-delivered partner therapy (PDPT).
c. Three randomized controlled trials, including one conducted in King County, WA, demonstrated that giving patients' medication to give to their sex partners decreases the risk that patients will become reinfected, and increases the proportion of partners that receive treatment.
d. Based on the findings of these trials, CDC incorporated EPT into the 2006 STD Treatment Guidelines. The WA State Department of Health has also developed guidelines for the use of EPT, and those guidelines have been endorsed by the WA State Board of Medical Quality Assurance, the state Pharmacy Board, and the WA State Medical Society.
e. The system being instituted was been in place in King County, WA since 2004.
f. A consortium comprised of the University of Washington, the State Department of Health, and Public Health – Seattle & King County subsequently received a National Institute of Health grant to evaluate an EPT system throughout the state. That grant is paying for the EPT program in which your pharmacy is collaborating.

B) Overview

Flow diagram of how prescriptions will be arranged for PDPT

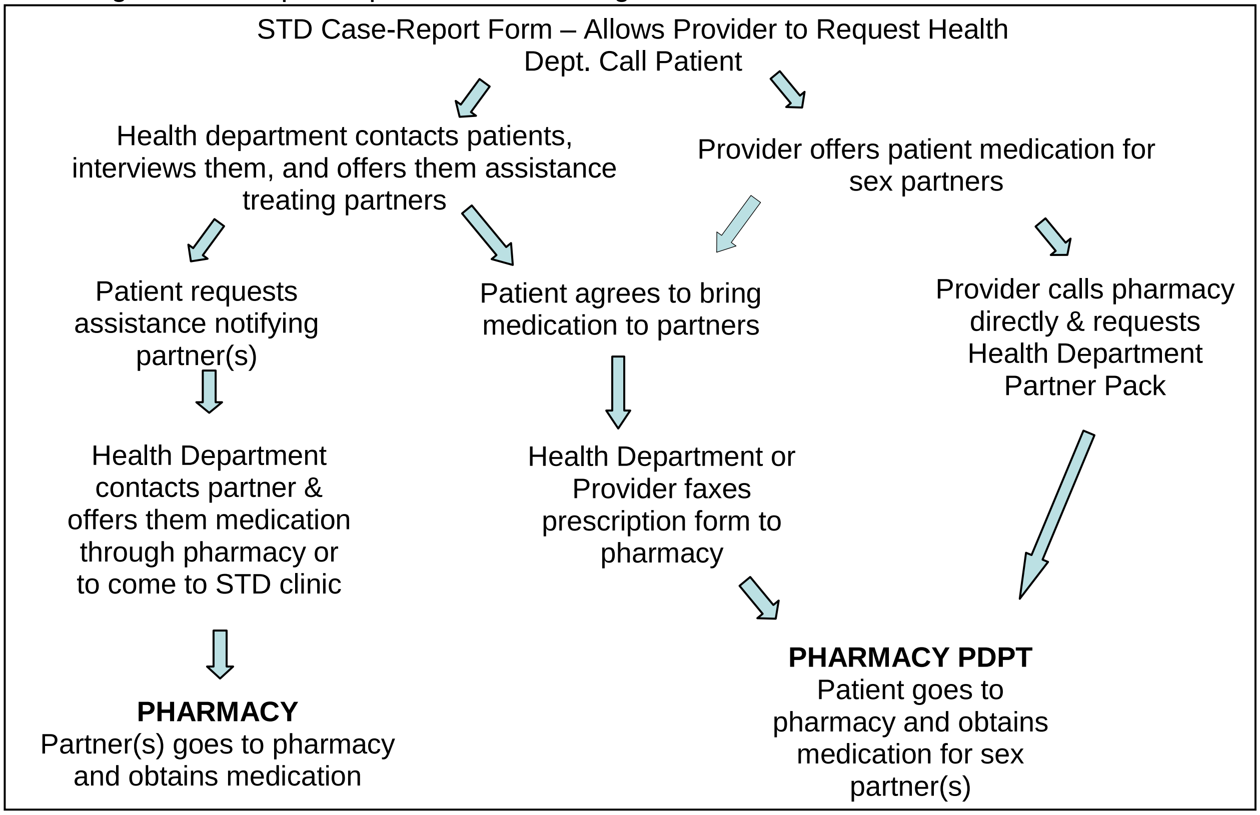

1. Medications distributed in “Partner Packs”.
    a. Partner Pack medications
        i. Chlamydia patients/partners (pack 1) - azithromycin 1g
        ii. Gonorrhea patients/partners (pack 2) – azithromycin 1g + cefixime 400mg
    b. Packs will contain printed instructions on
        i. How to take medications
        ii. A warning not to take medications if the partner has an allergy to penicillin or related drugs (GC packs) or azithromycin or another macrolide. Persons with allergies will be instructed to call the PHSKC STD clinic.
        iii. Advice to seek an evaluation for STD
        iv. Printed instructions will be sealed into the partner pack
    c. All prescriptions include instructions that child-proof packaging is not desired
    d. Partner packs are prepackaged by the PHSKC pharmacy and sealed.
2. Prescription Flow
    a. Source of Prescriptions and Mechanisms of Prescribing
        i. Prescriptions may come either from clinicians in the community or directly from the health department.
        ii. Prescriptions can either be faxed to the pharmacy or can be called into the pharmacy.
            1. Faxed prescriptions should arrive on a “Washington State Expedited Partner Therapy Program Prescription Form.” There is also a form attached to the WA State STD case report form. (Sample copies of both forms are in the forms section.)
            2. When clinicians telephone a prescription for EPT to a pharmacy, they will need to identify that they want to use public health medications.
        iii. Public health medications are meant for use for sex partners of persons diagnosed with gonorrhea or chlamydial infection. They are not meant primarily to treat persons initially diagnosed with an STD, though there may be some instances in which clinicians use them in this way.
    b. Prescription is filled
        i. The prescription will have only the first name, last name and a birthdate (if available) of the partner receiving medicine
        ii. The prescription should be held for three week for patient or partner to pick–up
        iii. Prescriptions are canceled and restocked after three weeks
3. Prescription Follow-up
    a. Pick up conformation - If a pharmacy had at least one prescription for EPT called/faxed/outstanding in any given week, the local health department will fax the “Washington State Expedited partner Therapy Program Prescription Pick-Up Confirmation” form (a sample copy is in the forms section) to the pharmacy once a week
        i. The form should be completed and faxed back to the local health department
        ii. The local health department will place reminder calls to all patients that have not picked up their prescriptions for up to three weeks before they are canceled and restocked

4. Data Entry Procedures - Data entry should be done according to pharmacy procedures and using the EPT DNC umbers
5. EPT Restocking Procedures
    a. EPT drug restocking- The "Washington State Expedited Partner Therapy Partner Pack Request Form" (a sample copy is in the forms section) should faxed to the EPT Coordinator, Cheryl Malinski, at (206) 744-2262 requesting the amount of drugs needed. The EPT packets will be expressed mailed
    b. Some pharmacy chains may develop restocking procedures using other distribution systems

PHSKC STD Clinic Policy: Patient Delivered Partner Therapy (PDPT) for gonorrhea & chlamydial infection

I) Candidates population for routine PDPT
   a. Criteria – patient should meet both criteria
      i. Gram stain (gonorrhea), culture or nucleic acid amplification test confirmed gonorrhea or chlamydial infection
      ii. Heterosexual
II) DIS vs. Clinician provided PDPT
   a. Patients should be referred to DIS or other partner notification staff if they meet one or more of the following 4 criteria
      i. >1 sex partner in preceding 2 months
      ii. ≥1 partner the patient states they do not plan to have sex with again
      iii. Patient wants assistance
      iv. Man who has sex with men
   b. Heterosexuals who meet the above criteria should be referred to Julija Rankis or Antelia Medina when they are available. If Julija or Antelia are not available, these patients should be referred to other DIS.
   c. Patients with none of the above criteria do not need to talk to the DIS and should be offered PDPT to be provided directly by clinicians
III) Procedures for clinician provided PDPT
   a. Inform all patients that we want to evaluate their partners at the clinic. The partner pack is given because partner referral often does not happen.
   b. Dispensing
      i. All PDPT should be dispensed in pre-packaged partner packs.
         1. Separate packs for gonorrhea & chlamydial infection
            a. Azithro for chlmydia
            b. Cefpodoxime + Azithro for GC
         2. Contains info about meds (allergy warning), STD, condoms
      ii. Limit of up to 3 packets
   c. Documentation on individual sheets for each partner and in chart
      i. Med room log – individual prescription forms stored in log
         1. Index patient name
         2. Partner name – intended recipient
   d. Document in patient chart that PDPT provided. Do not record partner name
IV) Legality - Approved by WA State Pharmacy Board, the WA State Board of Medical Quality Assurance, the WA State Department of Health and the WA State Medical Society
V) Use of PDPT in MSM
   a. Clinic policy is to NOT routinely offer MSM PDPT
      i. In one study that included patients seen in our clinic, 5.5% of MSM seeking care as contacts to gonorrhea or chlamydial infection were newly diagnosed with HIV
      ii. There are no published data evaluating PDPT in MSM
      iii. The balance of risk and benefits of PDPT in MSM in unknown.
      iv. We hope to soon start a randomized trial to evaluate PDPT in MSM.

v. Outside of the trial, PDPT should only be used in MSM under very rare circumstances, preferably after discussion with Matt Golden or Taraneh Shaffi.

PHSKC Family Planning Clinic Policy: Patient Delivered Partner Therapy (PDPT) for gonorrhea & chlamydial infection

VI) Candidate population for routine PDPT
  a. Gram stain (gonorrhea), culture or nucleic acid amplification test confirmed gonorrhea or chlamydial infection
  b. Heterosexual

VII) Inform all patients (index cases) that we want to evaluate their partners at the clinic. The partner pack is only given because partner referral does not happen.

VIII) Use the King County STD Case Report form to document the decision regarding how to treat partner(s). Complete all portions of the form. We are the health department and will be dispensing PDPT for most index cases agreeing to do this. Sometimes a private clinic patient may present to be treated by PHSKC and in that case they are to be seen as any other STD/FP client.

IX) Dispensing
  a. All PDPT should be dispensed in pre-packaged partner packs.
  b. Write into the index case chart “medications provided for partner”
  c. Do not put the partner’s name into the index case’s chart
  d. Complete the PDPT prescription form in the logbook in pharmacy.
  e. There must be a partner name to write on the prescription form.
  f. Dispense the PDPT to index case to give to partner
  g. Separate packs for gonorrhea & chlamydial infection
    i. Azithro for CT
    ii. Cefixime + Azithro for GC
    iii. Contains info about meds (allergy warning), STD, condoms
    iv. Do not open the package. It is be opened by the partner.

X) Limit of up to 3 packets or 3 partner prescriptions per index case

XI) Legality
  a. Approved by WA state pharmacy board and board of medical quality assurance.
  b. No HIPPA conflict given there is no partner name in index chart and the pharmacy log book is just to record medication dispensing.

XII) MSM – 5% of partners evaluated as contacts to STD are newly diagnosed with HIV when evaluated. Therefore these clients are to be referred to DIS (PHSKC STD clinic) and not given PDPT

XIII) Who to refer to DIS for additional counseling and assistance with partner contact/treatment
  a. Criteria for referral
    i. >1 sex partner in preceding 2 months
    ii. One of more partners patient states they do not plan to have sex with again
    iii. Patient wants assistance or state they are unable or unwilling to contact a partner
    iv. Men who have sex with men

b. You should still offer PDPT to heterosexuals with gonorrhea or chlamydial infection. However, because patients with the above criteria are most likely to need public health partner notification assistance, they should be referred to STD program DIS.
c. Inform persons with the above risk criteria to expect to be contacted by PHSKC DIS
