# Supplementary material for: Uptake and Population-Level Impact of Expedited Partner Therapy (EPT) on Chlamydia trachomatis and Neisseria gonorrhoeae: The Washington State Community-Level Randomized Trial of EPT
Source: PLoS Med. 2015 Jan 15;12(1):e1001777. doi: 10.1371/journal.pmed.1001777 (PMC4295847; doi:10.1371/journal.pmed.1001777)
Supplement: S2 Text — (DOCX) [file pmed.1001777.s002.docx]

Analysis Plan

**Title:** Washington State EPT community randomized trial

**Background and Introduction:** Gonorrhea and genital chlamydial infection continue to be major public health problems throughout the world, with stable or somewhat increasing levels of morbidity observed in the U.S. Expedited partner therapy (EPT), the practice of treating the sex partners of persons with curable sexually transmitted infections (STI) without the partners’ mandatory prior assessment by a clinician, has been evaluated in four recent randomized controlled trials and has been shown to decrease rates of persistent or recurrent gonorrhea and chlamydial infection and to increase the proportion of sex partners treated. However, the feasibility of widely instituting EPT is uncertain, and, like other public health measures to control STI, the intervention’s potential population-level impact is unknown. In this application, we propose a stepped-wedge community-level randomized controlled trial of EPT for gonorrhea and chlamydial infection. This trial will randomly assign the temporal order in which EPT is implemented in WA State health districts and will be used to assess the impact of EPT on the prevalence of chlamydial infection among women tested in clinics supported through the CDC Infertility Prevention Project (IPP), and the incidence of reported gonorrhea among women in WA State.

**Specific Aim:** To determine whether EPT for genital chlamydial infection and for gonorrhea decreases the prevalence of chlamydial infection and the incidence of gonorrhea in the population. We hypothesize that EPT will decrease the prevalence of chlamydial infection among women tested in clinics supported through the CDC Infertility Prevention Project (IPP), and the incidence of reported gonorrhea among women in WA State.

**Study Design:**

##### This study was designed as a stepped-wedge community-level randomized trial (i.e. the order in which the intervention is instituted is randomly assigned). The stepped-wedge community-level randomization design is able to evaluate the effectiveness of the EPT intervention while controlling for a possible secular trend in gonorrhea and chlamydial morbidity in Washington state. In this trial the intervention was initiated in 4 waves. The starting dates and counties randomized in each wave are shown in table 1. Dates of the start of baseline data collection are given in table 1a.

| Table 1. Dates of start of the intervention, by wave | | |
| --- | --- | --- |
|  |  | Date Started |
| Wave 1 | Pierce  Thurston  Lewis  Clark, Skamania, Klickitat  Kittitas  Whitman | Oct 1, 2007 |
| Wave 2 | Yakima  Kitsap  Clallam, Jefferson  Grays Harbor  Chelan, Douglas  Benton, Franklin | June 1, 2008 |
| Wave 3 | Snohomish  Whatcom  Skagit, San Juan  Mason  Okanogan  Walla Walla | Jan 15, 2009 |
| Wave 4 | Spokane  Cowlitz, Wahkiakum, Pacific  Island  Grant  Ferry, Stevens, Pend Oreille  Adams, Garfield, Lincoln, Asotin, Columbia | August 1, 2009 |

| Table 1a. (Date of start of baseline data collection) | | |
| --- | --- | --- |
|  |  | Date Started |
| Wave 1 | Pierce  Thurston  Lewis  Clark,  Skamania,  Klickitat  Kittitas  Whitman | 4/16/2007  4/13/2007  4/24/2007  4/9/2007  4/25/07  4/23/07  4/30/2007  5/1/2007 |
| Wave 2 | Yakima  Kitsap  Clallam, Jefferson  Grays Harbor  Chelan, Douglas  Benton, Franklin | 12/04/2007  12/12/2007  01/03/2008  12/17/2007  12/25/2007  11/23/2007 |
| Wave 3 | Snohomish  Whatcom  Skagit, San Juan  Mason  Okanogan  Walla Walla | 07/18/2008  06/25/2008  07/15/2008  07/10/2008  08/20/2009  08/08/2008 |
| Wave 4 | Spokane  Wahkiakum, Pacific  Cowlitz, Island  Grant  Ferry, Stevens, Pend Oreille (Did not participate)  Adams,  Garfield,  Lincoln,  Asotin,  Columbia | 03/23/2009  04/01/09  04/03/2009  04/02/2009  04/07/2009  03/21/09  04/07/009  03/03/09  04/17/09  5/18/09 |

##### The EPT intervention tested included use of a case-report form to triage persons at high-risk for PN failure for public health intervention, and promotion of community-wide no-cost PDPT. The uptake of the intervention was monitored by interviewing a random sample of persons reported with each STI about their partners’ treatment and whether they received PDPT from their provider. The study’s primary outcome is the prevalence of *Chlamydia trachomatis* in women tested in WA State IPP clinics and the incidence of gonorrhea in women, excluding persons presenting as contacts to gonorrhea. The study population includes all counties in Washington State other than King (where EPT has been previously implemented). Data for the study include IPP clinic records and reportable disease records for the period 2006 (one year prior to the start of the intervention) through 2010 as well as data from the interviews.

**Testable hypotheses:** After controlling for underlying time trends across the state and correlation within health districts, intervention periods will be associated with lower prevalence of Ct in 14-25 yo women attending IPP clinics (excluding women whose reason for visit is exposure to Ct)

**Variables:**

The two primary sources of data for these analyses are 1) the IPP database (IPP), which contains one record per woman-visit for all visits to IPP clinics between 206 – 2010. Variables included in this database are listed in table 2a; 2) the Washington State reportable disease database (RD), which contains one record per case of gonorrhea or Chlamydia for all cases reported between 2006 – 2010. Variables in this database are listed in table 2b; 3) the Interview database (IV), which contains information on (phone) interviews with a randomly selected subset of individuals in the RD database. These interviews were conducted to determine the proportion of individuals with Gc and/or Ct who received EPT over time (i.e. the degree of uptake of the intervention). Interviews were conducted from 4 – 6 months before the start of the intervention in each wave through the end of the study (see appendix A). Variables included in this database are listed in table 2c.

| Table 2a IPP data | | | | |
| --- | --- | --- | --- | --- |
| Names | Description | Type | Units | Role in study/ add’l info |
| ctlab | Ct lab test result, original | categorical | 1 = Unsat  2 = Negative Ct  3 = Positive Ct  4 = Equivocal Ct |  |
| ctlabc | Ct lab test result, recoded | categorical | 0 = negative Ct  1= positive Ct | **Primary outcome**;  if ctlab = 1 or 4, this variable is coded as missing |
| gclab | Gc lab test result, original | categorical | 1 = Unsat  5 = Negative Gc  6 = Positive Gc  7 = Equivocal Gc |  |
| gclabc | Gc lab test result, recoded | categorical | 0 = negative Gc  1= positive Gc | if gclab = 1, 7 or missing, this variable is coded as missing |
| sex | Sex of participant | Categorical | 1 = Female | Only women are included in the database |
| age | Age of participant | Continuous | Years |  |
| dtvst | Date of visit | Julian date |  |  |
| routine | These are all derived variables that give the reason(s) for the participants visit | categorical | 0 = no  1 = yes | Routine visit |
| expct |  |  |  | Exposed to Ct |
| expgc |  |  |  | Exposed to Gc |
| expoth |  |  |  | Exposed to Other STI |
| resrcnct |  |  |  | Rescreen Ct |
| rescrngc |  |  |  | Rescreen Gc |
| preg |  |  |  | Pregnancy-related |
| symps |  |  |  | Symptoms |
| facility_county_name | Name of the county in which this visit occurred | categorical |  | County names give as a string |
| hdist | Which of 24 health districts is this county in? | categorical | Coded 1 – 24 in order shown in table 1 | King county coded as missing |
| wave | Which wave is this county in? | categorical | Coded 1 – 4; see table 1 | King county coded as missing |
| time | Which time period is this observation in? | categorical | 0 = July – Sept, 2007  1 = Mar – May, 2008  2 = Oct. 15, 2008 – Jan. 14, 2009  3 = May – July, 2009  4 = June – Aug, 2010 | These are the outcome time periods for the primary analysis |
| treat | Treatment indicator | categorical | 0 = control  1 = intervention | A function of time and wave |
|  |  |  |  |  |

**Statistical Analysis:**

We will summarize demographic characteristics, reason for visit, sexual behavior and disease prevalence for IPP attendees aged 14 – 25 years, by wave and total, for the period 10/06 – 9/07 (the year prior to the start of the intervention in wave 1 communities). We will also include in this summary Gc and Ct rates (per 100,000 population) for this period from the RD database. See mock table A.

Based on the IV database we will summarize the response rate to the phone surveys and the proportion receiving EPT, by wave, as a function of time since start of the intervention. See mock table B.

We will conduct three key analyses to understand the effect of the intervention on Ct prevalence as measured at IPP sites. These three analyses are described below:

1. Intent to Treat (ITT) analysis – This is the primary analysis for the trial. We will use mixed effects models, clustering on health district and site within health district to fit the following model to analyze all IPP records for the periods corresponding to the variable “time” in table 2a:

$$log\left( p_{ijk} \right)= \mu+\beta_{1}T_{1}+\beta_{2}T_{2}+\beta_{3}T_{3}+\beta_{4}T_{4}+X_{ij}\theta$$

where p_ijk_ is the probability of Ct infection in individual k at time j in county i (variable ctlabc in table 2), T_j_ is a 0/1 indicator indicating whether this observation is in observation period j, and X_ij_ is a 0/1 indicator of whether the intervention had started in county i at time j. Y_ijk_ is a 0/1 indicator of Ct infection in individual ijk. The parameter µ may be interpreted as the log risk of Ct during the control period across all counties; β_j_ is the log risk ratio associated with time period j (relative to the control period); and θ is the log risk ratio associated with the intervention. The model will cluster on health district and site within district to account for sites that do not report cases over the entire study period. An estimate of exp(θ) (the odds ratio associated with the intervention) and a 95% CI will be provided. We will test the primary hypothesis

Ho: θ = 0

Ha: θ ≠ 0

using α = 0.05 (two-sided).

data <- data[data$sex==1 & data$age>=14 & data$age <=25 & data$hdist!=23,]

data$hdist <- as.factor(data$hdist)

data$site <- as.factor(data$site)

lmer(ct ~ treat + as.factor(time) + (1|hdist) + (1|hdist:site), family=binomial, link=log, data=data)

1. Per protocol (PP) analysis – This analysis incorporates measurable delays in ramping up EPT. The ITT analysis in (1) assumes that EPT is being “fully delivered” (i.e. at its long-term maximum rate) within the same time interval as when it was introduced within each wave. However, the reality is that the EPT took time to fully ramp up. Therefore, this per protocol analysis will use a mixed effects model clustering on county and site within county (as in 1) but, instead of a 0/1 indicator of commencement of the intervention in county *i* at time *j*, *X_ij_* will be a fraction representing the percentage of partners notified for EPT in county *i* at time *j*. The fraction of partners notified will be obtained from interviews with the partners referred for PN assistance. This analysis has the advantage over the ITT analysis of incorporating known delays in the implementation of the intervention into the analysis. *θ* retains its interpretation as the log relative risk associated with the full effect of the intervention. An estimate of exp(*θ*) (the risk ratio associated with 100% of partners receiving treatment) and a 95% CI will be provided. We will test the hypothesis

H_o_: *θ* = 0

H_a_: *θ* ≠ 0

using a two-sided *α* of 0.05.

1. PP analysis with lag – This analysis incorporates measureable delays in ramping up EPT and estimates delays in effect of the intervention after ramp up. Regardless of known delays in the implementation of the intervention, the intervention may not have its full effect within the study period (after full implementation). In this study, unknown delays in the effect of the intervention will be estimated in a two-step process as follows:
2. We will use GEEs to fit a model clustered by county,

$$log\left( p_{ijk} \right)= \mu+\beta_{1}T_{1}+\beta_{2}T_{2}+\beta_{3}T_{3}+\beta_{4}T_{4}+X_{ij0}\theta_{0}+X_{ij1}\theta_{1}+X_{ij2}\theta_{2}+X_{ij3}\theta_{3}\ldots$$

with β and T as in (1) and X_ijl_ equal to 1 if time j is l time intervals after the start of the intervention and 0 otherwise. This step will provide us with separate estimates of the intervention effect *l* time units after beginning the intervention. As in (2), the X_ijl_ may be set as fractions to account for known delays in implementation.

ii) To estimate the delay in the effect of the intervention and the full, long-term effect of the intervention, we will use a non-linear regression of the form

$\hat{\theta}_{l}=(1-e^{-\frac{l}{b}})\theta+\varepsilon_{l}$,

where $\hat{\theta}_{l}$represents the estimated log risk ratio associated with the intervention after *l* time units obtained in step (i), *b* represents the rate of the delay in the effect of the intervention, *θ* represents the long term effect or full log odds ratio associated with the intervention, and *ε_l_* is the distributional error in the effect estimates known to be asymptotically normal with variances that can be approximated from step (3i). If Σ denotes the variance-covariance matrix of, then $\mathop{\hat{\theta}}\limits\sim\sim MVN\left(\theta\mu_{b},\Sigma\right)$, where $\mu_{b}=\left(\begin{matrix} 1-e^{-\frac{0}{b}} & 1-e^{-\frac{1}{b}} & 1-e^{-\frac{2}{b}} & 1-e^{-\frac{3}{b}} \end{matrix}\right)^{'}$and a closed form expression for the maximum likelihood estimate (MLE) of *θ* is. There is no closed form expression for$\hat{b}$, the MLE of *b*, which is required for estimating *θ*. Therefore, *b* will be estimated using the Newton-Raphson iteration algorithm for numerical optimization. The algorithm makes use of the one-term Taylor expansion series approximation of the partial derivative of the log-likelihood with respect to *b* as follows:

Let $\dot{l}_{b}(\theta,b|\mathop{\hat{\theta}}\limits\sim$denote the partial derivative of the log-likelihood with respect to *b*, $\ddot{l}_{b}(\theta,b|\mathop{\hat{\theta}}\limits\sim$ denote the 2^nd^ partial derivative of the log-likelihood with respect to *b*, and $\hat{b}_{n}$denote the *n*^th^ approximation of$\hat{b}$. Then

$$\dot{l}_{b}={\theta\dot{\mu}}_{b}^{'}\Sigma^{-1}\left( \hat{\theta}- \theta\mu_{b} \right)$$

$$\ddot{l}_{b}={-\theta^{2}\dot{\mu}}_{b}^{'}\Sigma^{-1}\dot{\mu}_{b}+\ddot{\theta\mu}_{b}^{'}\Sigma^{-1}\left( \hat{\theta}- \theta\mu_{b} \right)$$

and

We start with an initial value, $\hat{b}_{0}$and successively update $\hat{\theta}$ and$\hat{b}$until convergence. An estimate of exp(*θ*) (the risk ratio associated with 100% of partners receiving treatment) and of *b* (the rate of delay in intervention effect) and their 95% CIs will be provided. Standard errors for constructing the CIs will be derived from the information matrix evaluated at the value of the estimates.

We will test the hypothesis

H_o_: *θ* = 0

H_a_: *θ* ≠ 0

using a Wald test and a two-sided *α* of 0.05.

**Dummy Tables and Figures:**

| Mock table 1. Demographics, Behavior and disease information during the pre-intervention period (Oct, 2006 – Sept, 2007) | | | | | |
| --- | --- | --- | --- | --- | --- |
|  | Wave 1 | Wave 2 | Wave 3 | Wave 4 | Total |
| Percent female |  |  |  |  |  |
| Age |  |  |  |  |  |
| Reason for visit:  Routine  Symptoms  STD Screening  Exposed to CT  Exposed to Other STI  Pregnancy-related  Rescreen |  |  |  |  |  |
| Race:  White  Black  Native American  Asian  Other  Multiple |  |  |  |  |  |
| Ethnicity:  Hispanic  Non-hispanic |  |  |  |  |  |
| Risk (% yes)  Ct in prev 12 mo  Other STD (12 mo)  > 2 partner (60 days)  New partner (60 days)  Sx partner (60 days)  Condom last sex |  |  |  |  |  |
| Ct prevalence (IPP) |  |  |  |  |  |
| Ct rate (RD) per 100,000 |  |  |  |  |  |
| Gc rate (RD) per 100,000 |  |  |  |  |  |

| **Mock table 2. Reasons for visit within each group of counties by time period, expressed as a percentage of total visits for those counties for that time period. Within each cell, the order is**  Routine  Symptoms  STD Screening  Exposed to CT  Exposed to Other STI  Pregnancy-related  Rescreen | | | | | |
| --- | --- | --- | --- | --- | --- |
|  | **Time** | | | | |
|  | **0** | **1** | **2** | **3** | **4** |
| **Wave 1** |  |  |  |  |  |
| **Wave 2** |  |  |  |  |  |
| **Wave 3** |  |  |  |  |  |
| **Wave 4** |  |  |  |  |  |

**Appendix A**

| Table 1. Date of start of interviews in each Health district | | |
| --- | --- | --- |
|  |  | Date Started |
| Wave 1 | Pierce  Thurston  Lewis  Clark,  Skamania,  Klickitat  Kittitas  Whitman | 4/16/2007  4/13/2007  4/24/2007  4/9/2007  4/25/2007  4/23/2007  4/30/2007  5/1/2007 |
| Wave 2 | Yakima  Kitsap  Clallam, Jefferson  Grays Harbor  Chelan, Douglas  Benton, Franklin | 12/04/2007  12/12/2007  01/03/2008  12/17/2007  12/25/2007  11/23/2007 |
| Wave 3 | Snohomish  Whatcom  Skagit, San Juan  Mason  Okanogan  Walla Walla | 07/18/2008  06/25/2008  07/15/2008  07/10/2008  08/20/2009  08/08/2008 |
| Wave 4 | Spokane  Cowlitz,  Wahkiakum,  Pacific  Island  Grant  Ferry, Stevens, Pend Oreille (Did not participate)  Adams,  Garfield,  Lincoln,  Asotin,  Columbia | 03/23/2009  04/03/2009  04/01/2009  04/01/2009  04/02/2009  04/07/2009  03/21/2009  04/07/2009  03/03/2009  04/17/2009  05/18/2009 |
