# Supplementary material for: Uptake and Population-Level Impact of Expedited Partner Therapy (EPT) on Chlamydia trachomatis and Neisseria gonorrhoeae: The Washington State Community-Level Randomized Trial of EPT
Source: PLoS Med. 2015 Jan 15;12(1):e1001777. doi: 10.1371/journal.pmed.1001777 (PMC4295847; doi:10.1371/journal.pmed.1001777)
Supplement: S3 Text — (PDF) [file pmed.1001777.s003.pdf]

HUMAN SUBJECTS STATUS REPORT – PLEASE RESPOND PROMPTLY

BOX FOR COMMITTEE USE ONLY
MASTER ☐ COMM. ☐ INVEST. ☐
06-1345-C 02
**APPLICATION NO.**

Matthew Golden, Asst Professor of Medicine
Dept. Internal Medicine, Division of Infectious Diseases
Box 359777
Telephone: (206) 731-6829
Fax (206) 731-4151
golden@u.washington.edu

Community-level RCT of Expedited Partner Therapy HSC #06-1345-C 01

Human subjects approval for this activity expires on ~~9/10/06.~~ 9/28/06 Please complete this form according to the instructions below and send it to the Human Subjects Division, Box 351412, at least **six weeks before the expiration date**. This Status Report form and other Human Subjects Division forms are available on our web site, http://depts.washington.edu/hsd. Please note: You may not recruit new human subjects or continue your activity with previously enrolled subjects unless you have active human subjects approval.

☐ **DO NOT RENEW**: Send **one** typed, completed and signed original Status Report form. Please note that you should maintain approval until the data analysis for this activity is complete.

X **RENEW**: Send **one** typed, completed and signed original and **eight** copies of the Status Report plus **nine** copies of each currently approved consent and assent form (and HIPAA authorization form, if you are using one). [**NOTE**: for Committee E applications (e.g. 99-0000-**E** 01), send **one original** and **one copy** of the Status Report and **two copies** of each consent and assent form you are currently using].

PLEASE NOTE: If you plan to propose changes or add new funding, send them under separate cover, not with the Status Report. Use the Modification form and submit it to the Human Subjects. You can download this form from our website at http://depts.washington.edu/hsd/formin.htm.

Status Report Checklist
- ☒ Nine single-sided copies* of Status Report, signed by the principal investigator
- ☐ Nine single-sided copies* of the currently approved consent and assent forms
- ☐ ***two copies only for Committee E applications** (extra copies are recycled)
- ☐ One copy of each manuscript since last approval, if applicable
- ☐ Status Report typed, not handwritten (Handwritten forms will be returned)
- ☐ Radiation Safety Committee (543-0463) renewal requested, if appropriate
- ☐ **All forms collated to create nine review packets**

RECEIVED
Human Subjects Division
AUG 16 2006
UW

I acknowledge that this Status Report represents an accurate and complete description of my research.

___Matthew Golden [signature] _8/11/06______
Typed name and original inked signature of principal investigator Date

BOX FOR COMMITTEE USE ONLY

Sara Webb / CF 9/14/2006 Approve ☒ Disapprove ☐
Human Subjects Review Committee Signature Date

Subject to the following conditions: ________________

Period of approval is one year, from 9/29/2006 through 9/28/2007

VALID ONLY AS LONG AS APPROVED PROCEDURES ARE FOLLOWED

Status Report Form

Version 4.5

W UNIVERSITY *of* WASHINGTON
Human Subjects Division
Box 359470
Seattle, WA 98195-9470
Phone: 206-543-0098
Fax: 206-543-9218

For definitions, see the second to last page. For instructions on how to complete this form, see the last page.

For HSD Office Use Only		Date Received:
[ ] Master Copy	[X] Approved	RECEIVED Human Subjects Division AUG 27 2013 UW
[ ] IRB Working Copy	[ ] Disapproved	
[ ] Researcher Copy	[ ] Withdrawn	
[ ] Full IRB Review Required		
[✓] Expedited Review		DORA CRR # 8

**Approval period from:**	SEP 30 2013	**To:**	SEP 29 2014
**Date of IRB action:**	SEP 05 2013	**Printed name:**	Kristin Puhl
**IRB Chair or Designee Signature:**	[signature]		
**Notes:**			

Research Study Information			
**Submission Reason**	[X] RENEW IRB application	[ ] CLOSE IRB application	
**Expiration date of IRB approval**		9/29/2013	
**IRB Application #**	**29923**	**IRB Committee**	G J
**IRB Application Title**	**Community-level RCT of expedited partner therapy**		
**Lead Researcher Name**	**Matthew Golden**	**Contact Name**	
Position and/or academic appointment	Professor, Director, Public Health - Seattle & King County HIV/STD Program	Position and/or academic appointment	
Department/Division	Medicine	Department/Division	
Phone #	206-744-6829	Phone #	
Fax #	206-744-4151	Fax #	
Campus Box #	359777	Campus Box #	
Street address, if applicable		Street address, if applicable	
Email	golden@u.washington.edu	Email	

[ ] Person completing this form is the same as the Lead Researcher	[ ] Person completing this form is the same as the Contact	
Name of Person Completing This Form (If not Lead Researcher or Contact):	Email:	Phone:

**Name and Mailing Address for all paper-based correspondence** (if blank, correspondence will be directed to contact person, or lead researcher if no contact person)		
Name:	Campus Box#:	Other address if not at UW:
