# Supplementary material for: Uptake and Population-Level Impact of Expedited Partner Therapy (EPT) on Chlamydia trachomatis and Neisseria gonorrhoeae: The Washington State Community-Level Randomized Trial of EPT
Source: PLoS Med. 2015 Jan 15;12(1):e1001777. doi: 10.1371/journal.pmed.1001777 (PMC4295847; doi:10.1371/journal.pmed.1001777)
Supplement: S4 Text — (DOC) [file pmed.1001777.s004.doc]

CONSORT 2010 Checklist if information to include in a cluster randomized trial

| **Section/topic and item No.** | **Item** | **Page no.** | **Comment** |
| --- | --- | --- | --- |
| **Title and abstract** |  |  |  |
| 1a | Identification of clustered randomized trial in title | Title page | We have used to term community-level randomized trial instead of cluster randomized trial as we believe this is more descriptive and specific for the time of clustered trial we conducted |
| 1b | Structured summary of trial design | Abstract |  |
| **Background and Objectives** |  |  |  |
| 2a | Rationale for cluster design | Page 6 |  |
| 2b | Whether objectives pertain to the cluster or individual | Page 6 |  |
| **Methods** |  |  |  |
| 3a | Definition of cluster and description of how the design features apply to clusters | Page 6-7 |  |
| 3b | Important changes to methods after trial commenced and reasons | Page 9 | We did not change trial methods after the study commenced. However, some aspects of the intervention changed after initiation of the study due to factors beyond the study’s control. |
| Participants |  |  |  |
| 4a | Eligibility criteria for clusters | Page 6 |  |
| 4b | Setting and locations where data were collected | Page 6 |  |
| Interventions |  |  |  |
| 5 | Whether interventions pertain to clusters or individual participants or both | Page 7 |  |
| Outcomes |  |  |  |
| 6a | Define outcomes and whether they pertain to individual, cluster or both | Page 10 |  |
| 6b | Changes in trial outcomes after trial commenced | Page 11 | A single post-hoc analysis is included looking at a combined gonorrhea or chlamydia outcome |
| Sample size |  |  |  |
| 7a | Method of calculation of clusters (whether equal or unequal cluster size assumed), cluster size, coefficient of correlation, indication of uncertainty | Page 11 |  |
| 7b | Interim analysis |  | None |
| Randomization |  |  |  |
| 8a | Method to generate randomization sequence | Page 7 |  |
| 8b | Details of stratification or matching if used | Page 7 |  |
| Allocation or concealment mechanism |  |  |  |
| 9 | Specification that allocation was based on clusters rather than individuals and whether allocation concealment (if any) was at the cluster level, individual participant level or both | Page 7 |  |
| Implementation |  |  |  |
| 10a | Who generated random allocation sequences, enrolled clusters, and assigned clusters to interventions | Page 7 |  |
| 10b | Mechanism by which individual participants were included in clusters for the purposes of the trial (such as complete enumeration, random sample) | Page 10 |  |
| 10c | From whom consent was sought (representatives of the cluster or individual cluster members, both, and whether consent was sought before or after randomization | Page 6 |  |
| Blinding |  |  |  |
| 11a | If done, who was blinded after assignment to interventions | Page 7 |  |
| 11b | Description of similarity of interventions |  | NA |
| **Results** |  |  |  |
| Participant flow diagram |  | Figure 1 |  |
| 13a | Number of clusters randomly assigned, receiving intended treatment and analyzed | Figure 1 |  |
| 13b | For each group, losses and exclusions for both clusters and individual cluster members | Figure 1 |  |
| Recruitment |  |  |  |
| 14a | Dates defining periods of recruitment and follow-up | Page 10 |  |
| 14b | Why trial was ended or stopped |  | NA |
| Baseline data |  |  |  |
| 15 | Baseline characteristics for the individual and clusters and individual levels | Table 1 |  |
| Numbers analyzed |  |  |  |
| 16 | For each group, number of clusters included in each analysis | Figure 1 |  |
| Outcomes and estimation |  |  |  |
| 17a | Results at the individual or cluster level as applicable and a coefficient of intracluster correlation for each primary outcome | Figure 3,4,5  Page 14-17 |  |
| 17b | Absolute and relative effect sizes | Page 14-17 |  |
| Ancillary analyses |  |  |  |
| 18 | Results of any analyses performed, including subgroup analyses and adjusted analyses, distinguishing prespecificed from exploratory | Page 15,  Table 3-4 |  |
| Harms |  |  |  |
| 19 | Harms and unintended effects | Page 17 |  |
| **Discussion** |  |  |  |
| Limitations |  |  |  |
| 20 | Limitations, potential bias, imprecision | Page 18-19,21-22 |  |
| Generalizability |  |  |  |
| 21 | Generalizability of clusters and/or individuals | Page 21-22 |  |
| Interpretation |  | Page 17,20-21, 22-23 |  |
|  | Interpretation consistent with results, balancing benefits and harms and considering other evidence |  |  |
| Other information |  |  |  |
| 23 | Registration number and name of trial registry | Page 14 |  |
| Protocol |  |  |  |
| 24 | Access to protocol | Attached with manuscript |  |
| Funding |  |  |  |
| 25 | Sources of funding and other support, role of funders | Title page and acknowledgement |  |
